# Increased lipid production by heterologous expression of AtWRI1 transcription factor in *Nannochloropsis salina*

**DOI:** 10.1186/s13068-017-0919-5

**Published:** 2017-10-10

**Authors:** Nam Kyu Kang, Eun Kyung Kim, Young Uk Kim, Bongsoo Lee, Won-Joong Jeong, Byeong-ryool Jeong, Yong Keun Chang

**Affiliations:** 1 Department of Chemical and Biomolecular Engineering, KAIST, 291, Daehak-ro, Yuseong-gu, Daejeon, 34141 Republic of Korea; 2 Advanced Biomass R&D Center, 291, Daehak-ro, Yuseong-gu, Daejeon, 34141 Republic of Korea; 3 Plant Systems Engineering Research Center, Korea Research Institute of Bioscience and Biotechnology (KRIBB), 125, Gwahak-ro, Yuseong-gu, Daejeon, 34141 Republic of Korea

**Keywords:** Microalgae, *Nannochloropsis salina*, Transcription factor, Wrinkled1, Biofuels, TF engineering

## Abstract

**Background:**

Genetic engineering of microalgae is necessary to produce economically feasible strains for biofuel production. Current efforts are focused on the manipulation of individual metabolic genes, but the outcomes are not sufficiently stable and/or efficient for large-scale production of biofuels and other materials. Transcription factors (TFs) are emerging as good alternatives for engineering of microalgae, not only to increase production of biomaterials but to enhance stress tolerance. Here, we investigated an AP2 type TF Wrinkled1 in *Arabidopsis* (AtWRI1) known as a key regulator of lipid biosynthesis in plants, and applied it to industrial microalgae, *Nannochloropsis salina*.

**Results:**

We expressed *AtWRI1* TF heterologously in *N. salina,* named NsAtWRI1, in an effort to re-enact its key regulatory function of lipid accumulation. Stable integration *AtWRI1* was confirmed by RESDA PCR, and its expression was confirmed by Western blotting using the FLAG tag. Characterizations of transformants revealed that the neutral and total lipid contents were greater in NsAtWRI1 transformants than in WT under both normal and stress conditions from day 8. Especially, total lipid contents were 36.5 and 44.7% higher in NsAtWRI1 2–3 than in WT under normal and osmotic stress condition, respectively. FAME contents of NsAtWRI1 2–3 were also increased compared to WT. As a result, FAME yield of NsAtWRI1 2–3 was increased to 768 mg/L/day, which was 64% higher than that of WT under the normal condition. We identified candidates of AtWRI1-regulated genes by searching for the presence of the AW-box in promoter regions, among which lipid metabolic genes were further analyzed by qRT-PCR. Overall, qRT-PCR results on day 1 indicated that AtWRI1 down-regulated *TAGL* and *DAGK*, and up-regulated *PPDK*, *LPL*, *LPGAT1*, and *PDH*, resulting in enhanced lipid production in NsAtWRI1 transformants from early growth phase.

**Conclusion:**

AtWRI1 TF regulated several genes involved in lipid synthesis in *N. salina*, resulting in enhancement of neutral lipid and FAME production. These findings suggest that heterologous expression of AtWRI1 TF can be utilized for efficient biofuel production in industrial microalgae.

**Electronic supplementary material:**

The online version of this article (doi:10.1186/s13068-017-0919-5) contains supplementary material, which is available to authorized users.

## Background

The declining availability of petroleum-based fuels and their environmental impact, including global warming, has increased interest in the use of biomass as an alternative energy source [1]. Microalgae are promising feedstocks for biofuel production, with advantages that include high photosynthetic efficiency and biomass production. These organisms can grow rapidly while fast consuming CO_2_ and accumulate large amounts of lipids, which are converted to biofuels. In contrast to land plants, microalgae do not require large areas of arable land for cultivation and do not compete with food sources [2, 3]. However, high production costs limit the commercialization of microalgal biofuels, which requires significant genetic improvements for maximum production of lipids [4–6].

Conventional genetic engineering has focused on metabolic engineering of individual enzymes to increase lipid production of microalgae. For example, overexpression of glycerol-3-phosphate acyltransferase (*GPAT*) and glycerol-3-phosphate dehydrogenase (*GPDH*) in the diatom *Phaeodactylum tricornutum* increased lipid contents by 1.6-fold compared with wild type (WT) [7, 8]. Moreover, antisense knockdown of pyruvate dehydrogenase kinase (*PDK*) increased neutral lipid content in *P. tricornutum* by 82% [9], and overexpression of diacylglycerol acyltransferase (DGAT) increased lipid content by twofold in *Scenedesmus obliquus* [10]. However, overexpression of other enzymes such as acetyl-CoA carboxylase (ACCase) and fatty acid synthase (FAS) failed to enhance lipid production in microalgae [11], probably due to difficulties in genetic engineering and/or complicated metabolic feedback mechanisms in microalgae [12].

It is thus necessary to develop alternative strategies to regulate multiple genes in a metabolic pathway. Transcription factor (TF) engineering has been employed in global regulation of gene expression for certain metabolic pathways in plants and other eukaryotes, and is now emerging as a novel approach for improving microalgae for biomaterial production [13, 14]. In an effort to understand regulatory network of lipid metabolism, various TFs affecting triacylglycerol (TAG) accumulation have been identified through multi-omics (transcriptomic, proteomic and metabolomic) studies in *Chlamydomonas* [15]. For example, nitrogen-responsive regulator (NRR) plays an important role in TAG accumulation under N starvation condition [16]. Recently, a phosphorus starvation response 1 (PSR1) TF has been identified as a global regulator of carbon storage metabolism. The mechanisms underlying lipid and starch biosynthesis were confirmed by assessing the downstream target genes using PSR1 null and overexpressing mutants [17, 18].

Heterologous expression of plant TFs can be employed effectively in microalgae as TF engineering. TFs have conserved domains and can play similar roles in plants and microalgae [19, 20], resulting in improved regulatory systems in microalgae by heterologous expression of plant TFs with well-characterized functions. For instance, Dof (DNA binding with one finger) TFs containing a C_2_C_2_-type zinc finger-like motif play similar roles in different organisms [21–23]. In particular, GmDof from the soybean (*Glycine max*) is involved in lipid synthesis, and heterologous expression of GmDof has been shown to increase lipid contents in *Arabidopsis* [24]. Similar effects have also been reported in *Chlorella* and *Chlamydomonas* [25, 26].

Wrinkled 1 (WRI1) is a master regulator of lipid accumulation in the seed of *Arabidopsis*. WRI1 is a member of APETALA2-ethylene responsive element-binding protein (AP2-EREBP) family, and is controlled by LEAFY COTYLEDON2 (*LEC2*) [27]. WRI1 in turn regulates fatty acid biosynthetic genes with promoters containing an AW-box sequence [CnTnG(n)_7_CG], such as *PKp*-*ß1, BCCP2,* and *KAS1*, in *Arabidopsis* [28]. Genetic engineering of WRI1 has shown that seed oil production can be increased by overexpression of WRI1 homologs in plants, while decreased the null mutants [29, 30]. These results indicate that WRI1 has a positive effect on lipid biosynthesis in plants.

*Nannochloropsis* spp. are considered industrial model strains for biofuels due to their rapid growth and high lipid content. Moreover, *Nannochloropsis* can produce high value products including eicosapentaenoic acid (EPA), a *ω*-3 long chain polyunsaturated fatty acid (PUFA), used in health supplements [31]. However, genetic manipulation is required to make *Nannochloropsis* economically feasible for biofuel production [32–36]. Genome and transcriptome data of *Nannochloropsis* spp. are available, enabling efficient genetic engineering of these microalgae [37–42]. Moreover, several attempts of genetic modification have been recently found to increase lipid contents in *Nannochloropsis* [43–45]. Above all, *Nannochloropsis* can be used for TF engineering: genomic analysis of TFs and TF binding sites (TFBSs) in *Nannochloropsis oceanica* has been carried out to facilitate prediction of the TFs related to lipid synthesis [46]. In addition, we reported overexpression of a bHLH TF in *Nannochloropsis salina* that showed enhanced fatty acid methyl esters (FAMEs) productivity [47].

In the present study, we conducted heterologous expression of *Arabidopsis thaliana* WRI1 (AtWRI1) in *N. salina* CCMP1776, and obtained *N. salina* transformants, named NsAtWRI1, with high lipid content. To understand the mechanism by which AtWRI1 enhanced lipid synthesis in *N. salina*, AtWRI1-regulated genes involved in lipid synthesis were identified based on the presence of the AW-box, and the mRNA expression levels of selected target genes were confirmed by quantitative real-time polymerase chain reaction (qRT-PCR). These results provided an excellent proof of concept for heterologous expression of key regulatory TF from plants to improve lipid production in industrial microalgae.

## Results

### Heterologous overexpression of AtWRI1 in *N. salina*

**Fig. 1:**
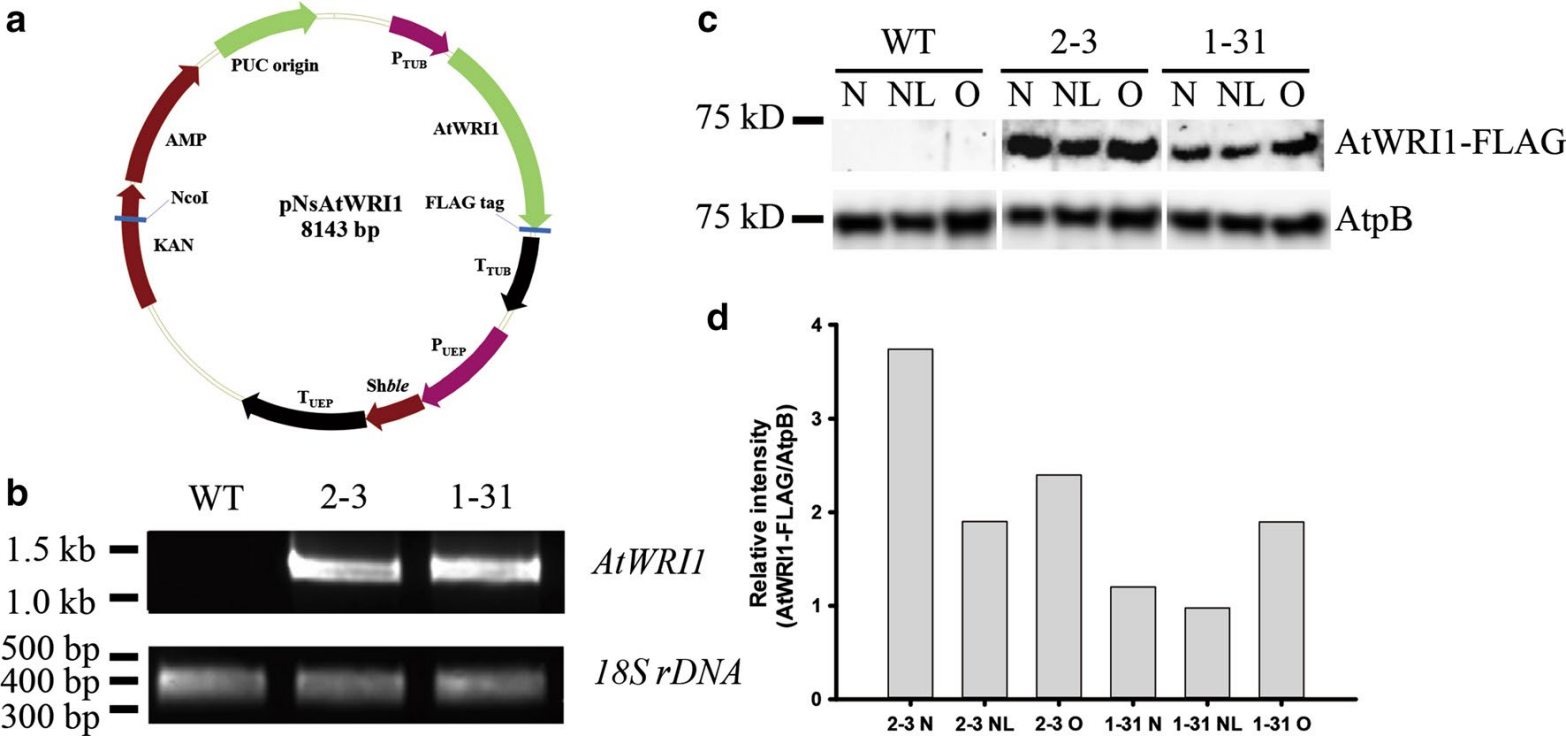
The pNsAtWRI1 vector and identification of the plasmid in the transformants. **a** Schematic map of the pNsAtWRI1 plasmid. **b** Detection of plasmids in NsAtWRI1 and WT. Genomic sequences of *AtWRI1* (1.3 kb) and *18S rDNA* (380 bp) were PCR amplified and the products were electrophoresed on agarose gels. **c** Western blotting of FLAG-tagged NsAtWRI1. The expected size of FLAG-tagged AtWRI1 was 49.4 kD, but running around 55 kD. AtpB (the CF_1_ *ß* subunit of ATP synthase of expected size 72.6 kD) was used as a loading control. **d** Relative intensity of AtWRI1-FLAG protein, which was calculated as the ratio of AtWRI1-FLAG vs AtpB. *WT* wild type, *N* normal conditions, *NL* nitrogen limitation, *O* osmotic stress

We constructed an AtWRI1 overexpressing vector, pNsAtWRI1 (Fig. 1a), and transferred linearized pNsAtWRI1 into *N. salina* CCMP1776 by particle bombardment. We obtained two transformants expressing the AtWRI1 protein, named NsAtWRI1 2–3 and 1–31. Genomic PCR was performed to check the presence of the plasmid DNA (Fig. 1b). The primers W1 and W2 were used to detect the *AtWRI1* gene, and the primers SR6 and SR9 were used to detect *18S rDNA* (Additional file 1: Table S1). The *18S rDNA* was present in both *N. salina* WT and NsAtWRI1 transformants. In contrast, the *AtWRI1* transgene was present in NsAtWRI1 transformants, but absent from WT, indicating that pNsAtWRI1 had been successfully transferred into the transformants. The integration site of transgene into genomic DNA was confirmed by restriction enzyme site-directed amplification (RESDA) PCR and homology search using genomic database of *N. gaditana* B-31 (http://www.Nannochloropsis.org/) (Additional file 2: Figure S1) [41]. The transgene was integrated near the beta-tubulin gene (homologous to Naga_10009g86 in *N. gaditana*) and a hypothetical gene (homologous to Naga_100450g4 in *N. gaditana*) in NsAtWRI1 2–3 and 1–31, respectively. Western blotting confirmed that AtWRI1 protein was expressed in both transformants (Fig. 1c). We used the FLAG tag that was attached to the C-terminus of AtWRI1. A specific band for AtWRI1 protein was present only in NsAtWRI1 transformants, and the F-type H-ATPase *ß* subunit (Atpß) that was used as a loading control. The expression level of AtWRI1-FLAG protein was higher in 2–3 than 1–31 under all culture conditions (Fig. 1d), suggesting that 2–3 may provide better phenotype than 1–31. Taken together, these results indicated that pNsAtWRI1 had been successfully integrated into the genome and stably expressed in the transformed cells.

We identified three endogenous proteins containing the AP2 domain in *N. salina*, named NsAP2-1, NsAP2-2, and NsAP2-3, and compared with that of AtWRI1 (Additional file 3: Figure S2). All three showed very low sequence identity based on sequence analyses including BLASTp and CLCbio Main Workbench (Additional file 4: Table S2), suggesting that the endogenous AP2 TFs are not true orthologs, and may not interfere with AtWRI1.

### Growth analysis of NsAtWRI1 transformants

**Fig. 2:**
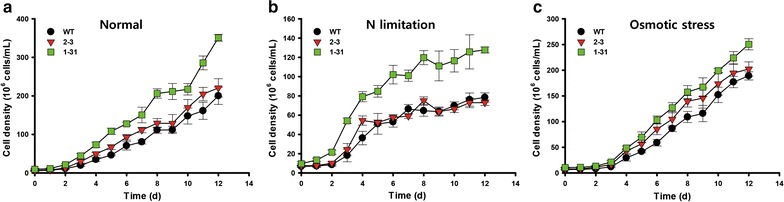
Growth of the NsAtWRI1 transformants under various culture conditions. Growth curve based on cell density under normal (**a**), N limitation (**b**), and osmotic stress conditions (**c**). The data points represent the average of samples and error bars indicate standard deviation (*n* = 4)

The phenotype of NsAtWRI1 transformants was analyzed under various culture conditions: normal, N limitation, and osmotic stress. The cell density of NsAtAWRI1 2–3 and 1–31 was higher than that of WT under normal and osmotic stress conditions (Fig. 2a, c). Under N limitation condition, the growth pattern and cell density of WT and 2–3 were similar, and 1–31 showed increased cell density (Fig. 2b). The maximum specific growth rate of NsAtWRI1 did not change significantly under normal and osmotic stress conditions. Although the specific growth rate was increased in NsAtWRI1 2–3 than in WT under N limitation, this difference did not affect their overall growth (Fig. 2b; Additional file 5: Table S3). Biomass yield of NsAtWRI1 transformants was similar or slightly increased compared to that of WT, indicating that AtWRI1 did not to affect growth significantly (Additional file 5: Table S3).

### Total lipid analysis of NsAtWRI1 transformants

**Fig. 3:**
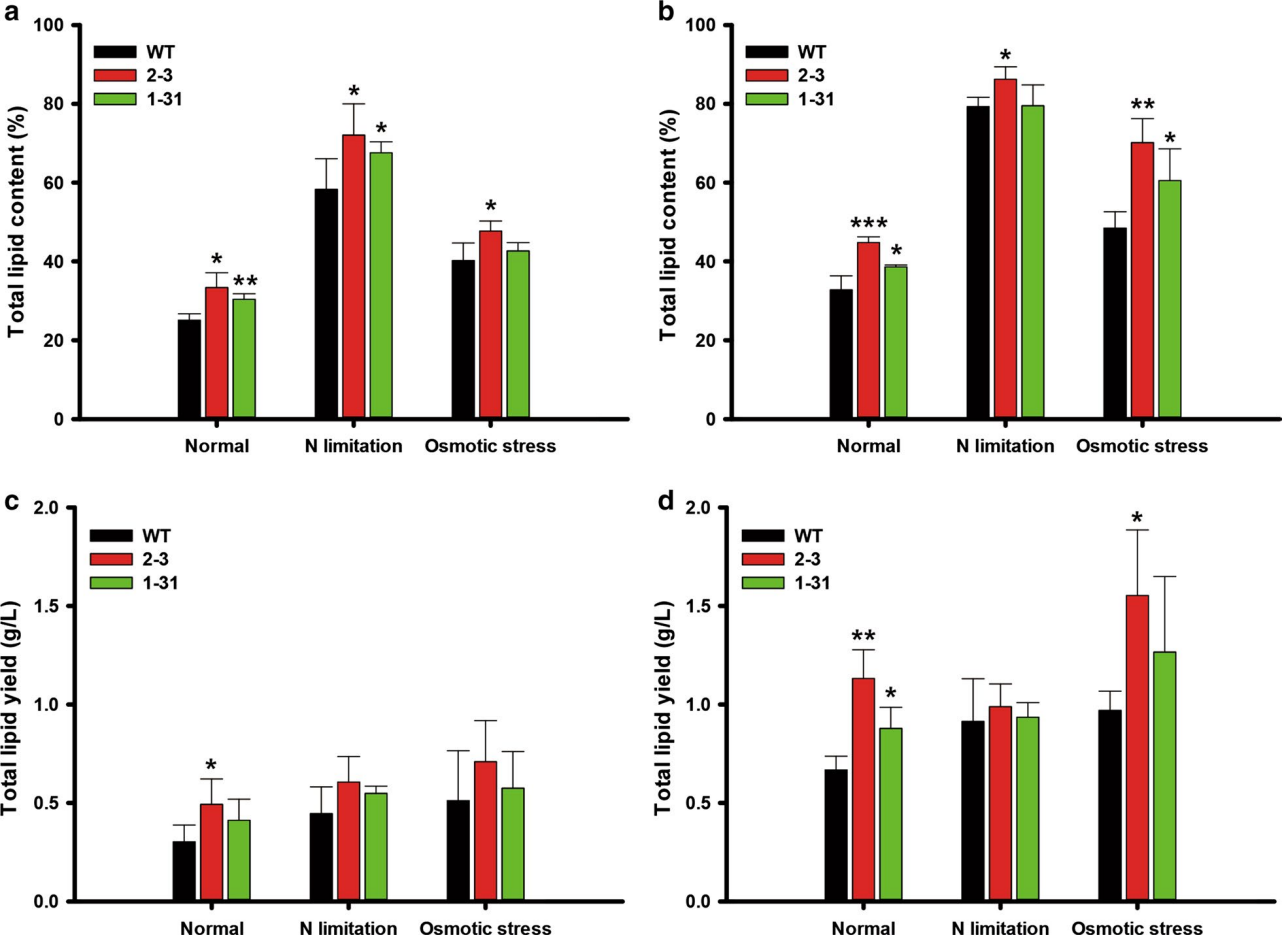
Total lipid analyses of NsAtWRI1 transformants under various culture conditions. Total lipid contents on day 8 (**a**) and day 12 (**b**). Total lipid yield on day 8 (**c**) and day 12 (**d**). The data points represent the average of samples and error bars indicate standard deviation (*n* = 4). Significant differences against WT for the same condition, as determined by Student’s *t* test, are indicated by asterisks (*P < 0.05, **P < 0.01, ***P < 0.001)

We analyzed total lipid content by employing gravimetric measurements (Fig. 3a, b). Total lipid contents of the transformants were increased on day 8 (Fig. 3a), which were 33 and 31% higher in NsAtWRI1 2–3 and 1–31 than in WT under the normal condition, respectively. NsAtWRI1 transformants also showed moderate increase in total lipids under N limitation and osmotic stress conditions. On day 12, transformants showed higher lipid contents under all culture conditions, and 2–3 showed higher increase in total lipids than 1–31 (Fig. 3b). Particularly, NsAtWRI1 2–3 showed 36.5 and 44.7% greater lipid contents compared to WT at day 12 under normal and osmotic stress conditions, respectively. However, under the N limitation condition, lipid contents were not significantly increased in NsAtWRI1 transformants compared with WT. This may be because lipid content was already saturated on day 12, regardless of WT and the transformants. We estimated total lipid yields by multiplying lipid contents and biomass yield as shown in Fig. 3c, d. Lipid yields were basically similar to lipid contents, because transformants did not show significant changes in the biomass. However, maximum increase in lipid yields was found in NsAtWRI1 2–3 showing approximately 70% increase under the normal condition on day 12 (Fig. 3d).

We also measured lipid composition on days 8 and 12 using HPLC that can separate lipids into neutral and polar lipids (Table 1). We identified hydrocarbons and TAG as neutral lipids, which were increased in NsAtWRI1 transformants under all conditions, but in general 2–3 was higher than 1–31, possibly reflecting higher expression levels of AtWRI-FLAG in 2–3 under all conditions than 1–31 (Fig. 1c, d). Membrane lipids (MGDG, DGDG, and PI) were also detected, but we did not find any substantial differences between transformants and WT, suggesting that AtWRI1 TF mainly affected accumulation of neutral lipids.

**Table 1:** Lipid composition (percentage of dry weight biomass, %) in WT and NsAtWRI1 transformants under different culture conditions analyzed by HPLC

Culture condition	Day	Strain	Neutral lipid (%)			Glycolipid (%)		Phospholipid (%)
			Hydrocarbon	TAG	Total^a^	MGDG	DGDG	PI
Normal	Day 8	WT	3.7 ± 2.3	1.0 ± 1.7	4.7 ± 3.2	19.3 ± 2.5	1.2 ± 1.4	n.d.
		2–3	7.1 ± 1.0*	4.8 ± 0.7*	11.9 ± 1.7*	21.5 ± 2.0	n.d.	n.d.
		1–31	1.8 ± 3.1	5.6 ± 0.7**	7.3 ± 2.4	20.4 ± 1.4	3.1 ± 0.3	n.d.
	Day 12	WT	2.8 ± 0.3	15.0 ± 2.3	17.8 ± 2.6	11.1 ± 1.5	3.1 ± 1.8	0.5 ± 0.0
		2–3	3.3 ± 0.3*	27.0 ± 2.9***	30.3 ± 2.8***	11.1 ± 1.2	3.3 ± 1.4	0.2 ± 0.2
		1–31	3.1 ± 0.2	18.6 ± 1.4*	21.6 ± 1.4*	11.0 ± 0.5	5.2 ± 0.4	0.8 ± 0.1
N limitation	Day 8	WT	5.8 ± 3.7	52.5 ± 6.9	58.3 ± 7.7	n.d.	n.d.	n.d.
		2–3	8.7 ± 1.3	63.4 ± 6.8*	72.0 ± 7.9*	n.d.	n.d.	n.d.
		1–31	9.3 ± 0.7	58.3 ± 2.4	67.6 ± 2.8*	n.d.	n.d.	n.d.
	Day 12	WT	6.6 ± 1.3	72.7 ± 3.4	79.3 ± 2.3	n.d.	n.d.	n.d.
		2–3	7.1 ± 0.3	79.1 ± 3.1*	86.2 ± 3.2*	n.d.	n.d.	n.d.
		1–31	5.7 ± 3.4	73.8 ± 6.3	79.5 ± 5.3	n.d.	n.d.	n.d.
High salt	Day 8	WT	10.9 ± 10.9	26.8 ± 7.6	37.7 ± 3.5	n.d.	2.6 ± 4.5	n.d.
		2–3	9.3 ± 11.6	31.7 ± 17.9	41.0 ± 14.1	n.d.	6.7 ± 11.7	n.d.
		1–31	20.5 ± 5.5	17.3 ± 4.3	37.8 ± 9.8	n.d.	4.9 ± 8.4	n.d.
	Day 12	WT	5.7 ± 0.9	34.5 ± 4.9	40.2 ± 4.7	3.9 ± 3.9	4.2 ± 1.4	0.2 ± 0.4
		2–3	6.7 ± 0.6	56.9 ± 5.7**	63.6 ± 6.2**	4.4 ± 4.4	2.1 ± 2.8	n.d.
		1–31	6.4 ± 0.9	45.9 ± 11.0	52.2 ± 11.7	4.5 ± 4.5	3.6 ± 1.5	0.2 ± 0.4

Analyses of lipid composition were performed using cells on day 8 and day 12. The data points represent the average of samples and error bars indicate standard deviation (*n* = 4). Significant differences against WT for the same condition, as determined by Student’s *t* test, are indicated by asterisks (*P < 0.05, **P < 0.01, ***P < 0.001)

*TAG* triacylglycerol, *MGDG* monogalactosyldiacylglycerol, *DGDG* digalactosyldiacylglycerol, *PI* phosphatidylinositol, *n.d.* not detected

^a^Total neutral lipid contents were calculated by sum of hydrocarbon and TAG contents

### FAME analysis of NsAtWRI1 transformants

**Fig. 4:**
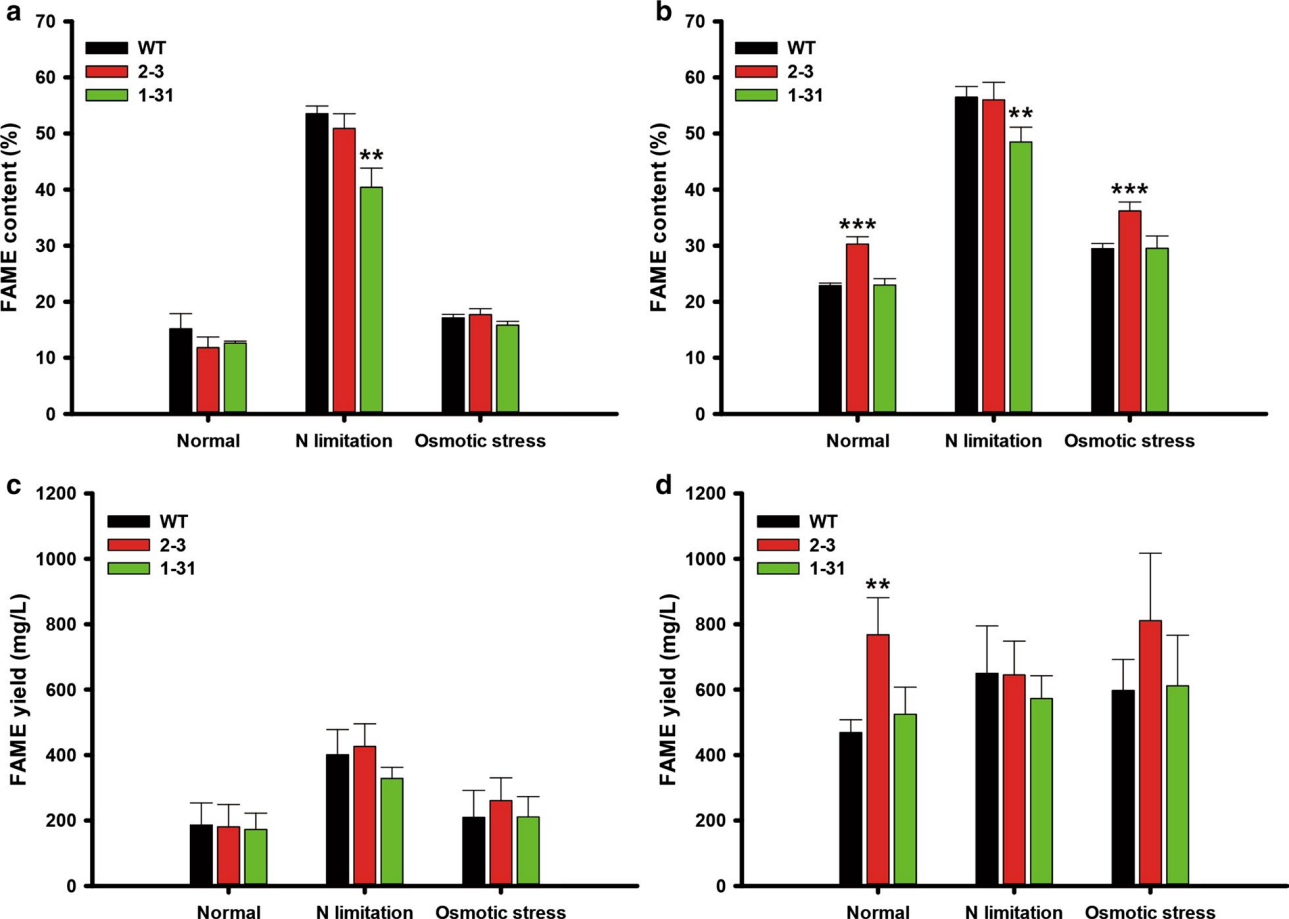
FAME quantitation of NsAtWRI1 transformants under various culture conditions. FAME contents on day 8 (**a**) and day 12 (**b**). FAME yield on day 8 (**c**) and day 12 (**d**). The data points represent the average of samples and error bars indicate standard deviation (*n* = 4). Significant differences against WT for the same condition, as determined by Student’s *t* test, are indicated by asterisks (*P < 0.05, **P < 0.01, ***P < 0.001)

To evaluate whether NsAtWRI1 transformants can be used practically for biodiesel production, we examined their FAME contents and yields (Fig. 4). The FAME content of NsAtWRI1 transformants and WT was not enhanced on day 8, regardless of culture conditions (Fig. 4a). In contrast, the FAME content of NsAtWRI1 2–3 was 32 and 22% greater than WT on day 12 under normal and osmotic stress conditions, respectively (Fig. 4b). Under the N limitation condition, FAME contents of NsAtWRI1 2–3 did not increase compared to WT, possibly because FAME contents were already saturated on day 8. Although FAME yield, calculated by multiplying biomass yield and FAME content, was similar in all samples on day 8 (Fig. 4c), NsAtWRI1 2–3 was 64% higher than WT under normal condition on day 12 (Fig. 4d). Increased total lipid contents by AtWRI1 TF appeared to affect FAME production (Figs. 3, 4). Improvement of FAME yield limited to the normal condition may reflect the original functions of AtWRI1 in plants, which will be discussed further. Overall, NsAtWRI1 2–3 behaved better in lipid accumulation than 1–31, and we thus chose NsAtWRI1 2–3 for further analyses.

### Identification and molecular analysis of AtWRI1-regulated candidate genes involved in lipid synthesis

To determine genes targeted by AtWRI1, we used matrix-scan provided by regulatory sequence analysis tool (RSAT), and obtained 475 genes with promoters containing an AW-box [48]. These 475 genes were classified by the gene ontology (GO) term enrichment test using Blast2GO software (https://www.blast2go.com/) into three domains: biological processes, cellular components, and molecular function (Additional file 6: Figure S3). Genes containing the AW-box represented a wide variety of functions. We noticed that many metabolic processes were included. Carbon utilization, lipid transport and localization processes were overrepresented compared with *N. salina* reference GO. These characteristics can be interpreted that many AW-box genes were involved in carbon and lipid metabolism. As lipid content was likely to be altered by AtWRI1 TF, we chose seven *N. salina* genes that may be involved in TAG and fatty acid synthesis (Table 2). These included genes encoding triacylglycerol lipase (*TAGL*) and lysophospholipase (*LPL*) that are involved in organic substance metabolic process; diacylglycerol kinase (*DAGK*) involved in phospholipid catabolic process; diacylglycerol acyltransferase family protein (*DGAT*) and lysophosphatidylglycerol acyltransferase 1 (*LPGAT1*) involved in transferring acyl groups; and pyruvate phosphate dikinase (*PPDK*) localized in chloroplasts and the dihydrolipoyllysine-residue acetyltransferase component of mitochondrial pyruvate dehydrogenase (*PDH*), which are involved in pyruvate metabolism. Gene structure and location of the AW-box are summarized in Additional file 7: Figure S4, and the lipid synthesis pathway including roles of the selected AtWRI1-regulated genes is presented in Additional file 8: Figure S5.

**Table 2:** *N. salina* lipid synthesis-related genes with promoters containing AW-boxes

Gene	Abb.	Gene ID^a^	Protein ID^a^	GO Names (ID) list^b^
Triacylglycerol lipase	*TAGL*	Naga_100057g25	EWM23354.1	P: organic substance metabolic process (GO:0071704)
Diacylglycerol kinase	*DAGK*	Naga_100089g7	EWM25228.1	P: phospholipid catabolic process (GO:0009395); P: organic substance metabolic process (GO:0071704); F: transferase activity (GO:0016740)
Acly transferase/acyl hydrolase/lysophopholipase	*LPL*	Naga_100156g4	EWM20908.1	P: organic substance metabolic process (GO:0071704)
Acyl-lysophosphatidylglycerol acyltransferase 1	*LPGAT1*	Naga_100501g5	EWM28984.1	F: transferase activity, transferring acyl groups (GO:0016746); F: transferase activity (GO:0016740)
Diacylglycerol acyltransferase family protein	*DGAT*	Naga_100028g44	EWM29372.1	F: transferase activity, transferring acyl groups (GO:0016746); F: transferase activity, transferring acyl groups other than amino-acyl groups (GO:0016747); F: transferase activity (GO:0016747)
Dihydrolipoyllysine-residue acetyltransferase component of pyruvate dehydrogenase mitochondrial-like	*PDH*	Naga_100006g108	EWM29544.1	P: pyruvate metabolic process (GO:0006090); P: organic substance metabolic process (GO:0071704); F: transferase activity (GO:0016740); C: cytoplasm (GO:0005737); C: organelle (GO:0043226); C: mitochondrial matrix (GO:0005759); C: cytoplasmic part (GO:0044444); C: intracellular organelle (GO:0043229)
Pyruvate phosphate dikinase	*PPDK*	Naga_100043g42	EWM23135.1	P: carbon utilization (GO:0015976); P: pyruvate metabolic process (GO:0006090); P: organic substance metabolic process (GO:0071704); F: transferase activity (GO:0016740)

^a^As *N. salina* CCMP1776 has not been annotated in NCBI, Gene ID and Protein ID refer to those of *N. gaditana B*-*31*

^b^GO enrichment test was performed using Blast2GO software with Fisher’s exact test and *P* value < 0.09

**Fig. 5:**
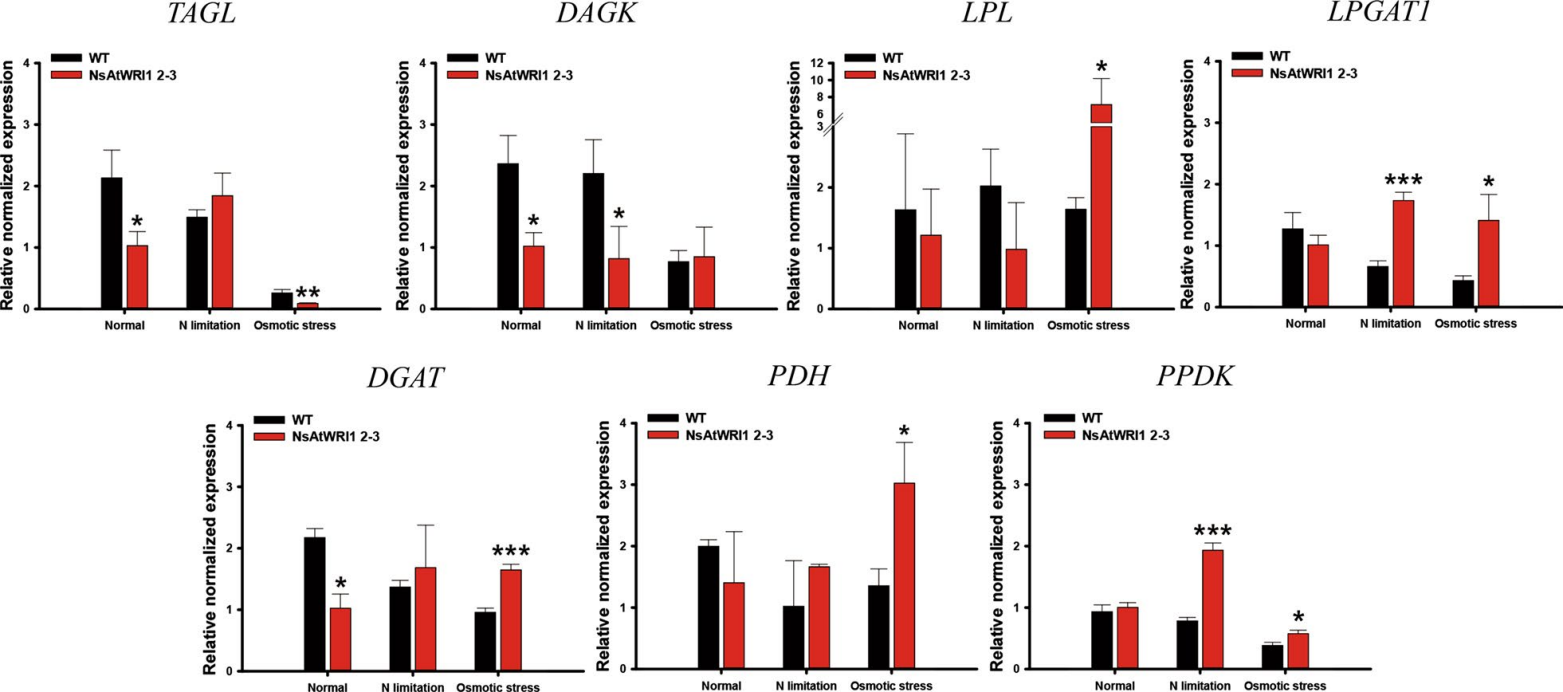
Expression profiles of AtWRI1-regulated candidate genes involved in lipid synthesis in NsAtWRI1 2–3. mRNA was obtained from cells which were incubated for 1 day under normal, N limitation and osmotic stress conditions. The expression levels of these genes were determined by qRT-PCR, normalized by that of *actin*. The data points represent the average of samples and error bars indicate standard deviation (*n* = 3). Significant differences against WT for the same condition, as determined by Student’s *t* test, are indicated by asterisks (*P < 0.05, **P < 0.01, ***P < 0.001). *TAGL* triacylglycerol lipase, *DAGK* diacylglycerol kinase, *LPL* lysophospholipase, *LPGAT1* lysophosphatidylglycerol acyltransferase 1, *DGAT* diacylglycerol acyltransferase family protein, *PPDK* pyruvate phosphate dikinase, *PDH* dihydrolipoyllysine-residue acetyltransferase component of pyruvate dehydrogenase mitochondrial-like

The mRNA expression levels of the seven lipid-related genes were analyzed by qRT-PCR (Fig. 5). The mRNA was obtained after cells were incubated for 24 h under normal, N limitation, and osmotic stress condition, and the endogenous actin gene was used for normalization. The *TAGL* expression level was lower in NsAtWRI1 2–3 than in WT under normal and osmotic stress conditions. The *DAGK* expression level of the transformant also decreased under normal and N limitation condition compared with WT. On the other hand, the expression level of *LPL* was greater in the transformant than in WT under osmotic stress conditions. The *LPGAT1* expression level of the transformant was also considerably higher than that of WT under N limitation and osmotic stress condition. The *DGAT* expression level was lower in the transformant than in WT under normal conditions, but higher under osmotic stress conditions. The *PDH* expression level of the transformant was higher than that of WT under osmotic stress condition, and the *PPDK* expression level of the transformant was higher compared to that of WT under N limitation and osmotic stress conditions. Overall, these up- and down-regulation expression patterns could be reconciled by their possible contribution to lipid production from early growth phase, which led us to classify the target genes into positive and negative groups, respectively. Significance of the classification will be discussed further.

We also performed an extended qRT-PCR to reveal expression profiles on days 0, 1, and 8 (Additional file 9: Figure S6). Expression of *LPL, PDH* and *PPDK* remained high in NsAtWRI1 2–3 compared to WT on day 8. Expression of *DGAT* was increased under the normal condition on day 8, which may have contributed to the increased lipid accumulation on day 12 under the normal condition (Fig. 3b, d). The expression pattern of DAGK was similar on day 8 compared to that of day 1. However, expression patterns of *TAGL* were changed on day 8, where they showed higher expression in NsAtWRI1 2–3 than WT. It is not certain why these genes showed reversed expression pattern on day 8, but it appears that these genes might have contributed less significantly to lipid accumulation on day 12.

## Discussion

Transcription factors’ engineering has emerged as an excellent strategy for improved productivity of biomass and lipids in plants, and is starting to be employed in microalgae. As manipulation of individual metabolic enzymes in microalgae may not be successful to enhance production of lipids effectively, global regulation of multi enzyme in lipid metabolic pathways can be emphasized [13]. In an effort to improve industrial microalgae for lipid production employing TF engineering, we chose AtWRI1 that is a key regulatory TF of lipid accumulation in *Arabidopsis* seeds [49]. Effects of this heterologous expression were positive on lipid content and yield, and we looked into revealing mechanisms for the enhancement. We identified its possible target genes by scanning the genome of *N. salina* for the presence of the AW-box containing promoters. Among the 475 genes, we selected seven genes involved in the lipid metabolism. We divided the seven genes into two groups: positive and negative ones based on their known functions, and their expression patterns were analyzed by qRT-PCR on day 1 (Fig. 5).

The negative group included TAGL and DAGK: TAGL degrades TAGs into diacylglycerol (DAG) and free fatty acids (FFA) [50], and DAGK catalyzes the phosphorylation of DAG leading to the formation of phosphatidic acid (PA) and indirectly decrease of TAG (Additional file 8: Figure S5) [51, 52]. It has been reported that down-regulation of TAGL results in TAG accumulation [50], and *DAGK* expression is lowered when a large amount of lipid is accumulated in microalgae [51]. NsAtWRI1 2–3 showed lower mRNA expression of *TAGL* under normal and osmotic stress condition, compared to WT. The *DAGK* expression level of NsAtWRI1 2–3 was also lower than that of WT under normal and N limitation. The down-regulation of *TAGL* and *DAGK* may contribute to accumulation of lipids under normal and stress conditions (Figs. 3, 5 and Table 1).

We classified PPDK, LPL, LPGAT1, and PDH into the positive group. Chloroplast PPDK plays an important role in the C_4_-like carbon-concentrating mechanism in addition to its classical role in reverse glycolytic pathway [53, 54]. As *Nannochloropsis* has C_4_-like carbon-concentrating mechanisms, high expression of chloroplast PPDK in NsAtWRI1 transformants may increase carbon fixation leading to increased lipid content [39]. LPL metabolizes lysophosphatidylcholine (LPC), producing acyl groups that can contribute to TAG accumulation. LPGAT1 converts lysophosphatidylglycerol (LPG) to phosphatidylglycerol (PG), which is incorporated into cellular membranes of microalgae [55]. As fatty acid in membrane phospholipid such as PG can be used for TAG synthesis by remodeling [56], LPGAT1 enzyme can indirectly contribute to increase neutral lipid. In addition, mitochondrial PDH catalyzes the conversion of pyruvate to acetyl-CoA, which can be used for fatty acid and neutral lipid synthesis [9]. In this study, the *LPL* and *PDH* expression level was higher in NsAtWRI1 than in WT under osmotic stress condition, and the expression level of *LPGAT1* was higher in NsAtWRI1 2–3 than in WT under N limitation and osmotic stress conditions. The increased expression level of *LPL*, *PDH*, and *LPGAT1* was likely to enhance the neutral lipid content (Figs. 3, 5 and Table 1), as summarized in schematic metabolic pathways relevant to these genes (Additional file 8: Figure S5).

The above candidates showed expression patterns consistent with their functional annotations. However, the *DGAT* gene that contained an AW-box did not show consistent expression pattern that varies depending on culture conditions on day 1. Whereas the mRNA expression level of *DGAT* was higher in NsAtWRI1 than in WT under osmotic stress condition, the *DGAT* expression level of NsAtWRI1 was lower than that of WT under normal condition. It is likely that this *DGAT* gene may not contribute to the lipid accumulation observed in NsAtWRI1. Other genes described earlier might be more important for the phenotypes in NsAtWRI1. These expression patterns indicate that NsAtWRI1 induces lipid accumulation by regulating the possible target genes from day 1, resulting in early lipid accumulation from day 8 (Fig. 3).

We also analyzed the expression profiles of the AtWRI1-regulated genes with days 0, 1 and 8. Expression of *LPL*, *PDH*, and *PPDK* on day 8 was increased in the NsAtWRI1 transformant, consistent with that on day 1, which may contribute to the increased lipid accumulation. We found that the expression pattern of *DAGK* was similar on day 8 compared to day 1, and that of *TAGL* was reversed. We are not certain of the significance of this reversal of gene expression for lipid metabolism. We speculate that the lipid accumulation would be combined effects of complex regulation of metabolic genes by the heterologous expression of AtWRI1 and endogenous regulatory mechanisms in *Nannochloropsis* under different culture conditions and times. We showed that the target genes were regulated more predictably early on day 1, and became up-regulated later on day 8 in general, suggesting that the early regulation contributed the lipid accumulation from early growth phase, resulting in increased lipid contents from day 8 (Fig. 3).

Based on these results, we suggest that heterologous expression of AtWRI1 is applicable to industrial production of biofuels. AtWRI1 2–3 expressing more AtWRI1 TF accumulated more neural lipids. Total lipid yield of NsAtWRI1 2–3 was increased by 1.1 mg/L, which is about 70% greater than WT under normal condition (Fig. 3d). Moreover, FAME yield, which can be used for biodiesel, of NsAtWRI1 2–3 was improved under the normal condition, where it reached as high as 768 mg/L, about 64% greater than in WT (Fig. 4d). This improvement may reflect the original function of AtWRI1 in *Arabidopsis*, where it is involved in lipid accumulation in seeds in response to developmental cues [28]. It is important to achieve improved lipid production under normal conditions for industrial production, because it is costly to add additional process in large-scale cultivation schemes. We showed that AtWRI1 was capable of regulating certain lipid metabolic genes and increasing lipid production in *N. salina*. Taken together, our results provided proof of concept that heterologous expression of plant key regulatory gene can be a strategy to improve industrial microalgae for production of biofuels and other materials.

## Conclusion

We demonstrated that heterologous expression of AtWRI1 TF could increase lipid contents and yield in *N. salina*. AtWRI1 seems to affect TAG synthesis and total lipid contents in *N. salina*. Total lipid content and yield of NsAtWRI1 started to increase on day 8. FAME yield of NsAtWRI1 2–3 was also 64% higher than that of WT on day 12 under normal condition. We identified seven possible AtWRI1-regulated genes involved in lipid metabolism in *N. salina* based on the presence of the AW-box in the promoter region, and analyzed their expression levels. In general, *DAGK* and *TAGL* were down-regulated, and *LPL*, *LPGAT1*, *PDH*, and *PPDK* were up-regulated, resulting in positive effects on lipid synthesis from early growth stage. This study provides proof of the concept that heterologous expression of AtWRI1 TF can be used for lipid production in industrial microalgae.

## Methods

### Microalgal strains and culture conditions

*Nannochloropsis salina* CCMP 1776 (Culture Collection of Marine Phytoplankton; now managed by the National Center for Marine Algae and Microbiota) was maintained in 250 mL Erlenmeyer baffled flasks containing 200 mL modified F2N medium [47], composed of 15 g/L sea salt (Sigma-Aldrich, USA), 10 mM Tris–HCl (pH 7.6), 427.5 mg/L NaNO_3_, 30 mg/L NaH_2_PO_4_∙2H_2_O, 5 mL/L trace metal mixture (4.36 g/L Na_2_ EDTA∙2H_2_O, 3.15 g/L FeCl_3_∙6H_2_O, 10 mg/L CoCl_2_∙6H_2_O, 22 mg/L ZnSO4∙7H_2_O, 180 mg/L MnCl_2_∙4H_2_O, 9.8 mg/L CuSO4∙5H_2_O, 6.3 mg/L Na_2_MoO_4_∙2H_2_O), and 2.5 mL/L vitamin stock (1 mg/L vitamin B_12_, 1 mg/L biotin, 200 mg/L thiamine∙HCl). The cells were incubated at 25 °C with agitation at 120 rpm under 120 µmol photons/m^2^/s of fluorescent light, while being supplied directly with air containing 2% CO_2_ at 0.5 vvm (volume gas per volume medium per minute). Normal medium in cultivation experiments was identical to modified F2N medium; in N limitation medium, the NaNO_3_ concentration was 75 mg/L, whereas in osmotic stress medium, the sea salt concentration was 50 g/L.

### Vector construction

The *AtWRI1* gene from *Arabidopsis* was cloned into a plasmid, named pNsAtWRI1 (Fig. 1a). To optimize *AtWRI1* expression in *N. salina*, codons were optimized based on the codon frequency of *Nannochloropsis* [57] and adjustment of GC contents below 60%. The sequence was synthesized by BIONEER Co. (South Korea). The coding sequence of AtWRI1 was PCR amplified using the W1 (forward) and W2 (reverse) primers (Additional file 1: Table S1), and the pNsAtWRI1 backbone was amplified using the W3 (forward) and W4 (reverse) primers. These two PCR products were combined by the Gibson assembly technique [58]. In the plasmid pNsAtWRI1, the codon-optimized *AtWRI1* gene was expressed under the control of the endogenous TUB promoter and terminator. The Sh*ble* gene was used for selection, which confers resistance to Zeocin, expressed under the control of the UEP promoter and terminator (Fig. 1a) [47].

### Particle bombardment transformation

The pNsAtWRI1 plasmid was linearized by digestion with NcoI and concentrated to 1 μg/μL for particle bombardment. The linearized plasmid was coated onto 0.6 μm microcarrier gold particles (Bio-Rad, USA) by mixing 3 mg of gold particles in 50 μL of 50% glycerol, 5 μL of the DNA, 50 μL of 2.5 M CaCl_2_, and 20 μL of 0.1 M spermidine. The resulting DNA-coated particles were suspended in 60 μL of 100% ethanol, and 12 μL was used for each bombardment transformation [47].

*Nannochloropsis salina* WT cells cultivated under normal condition were harvested in mid-exponential phase. The cells were concentrated, and 10^8^ cells on cellulose acetate membrane filters (Sartorius Stedim Biotech, Germany) were placed on modified F2N agar medium. Particle bombardment transformation was performed using a GDS-80 low-pressure gene delivery system (Wealtec, USA), with 625 µg of coated gold particles being shot in 700 psi helium, and with a 3 cm target distance between the muzzle of the gene gun and the cells. After transformation, the cells were allowed to recover by incubation for 1 day in modified F2N broth medium at 25 °C with dim light (5 µmol photons/m^2^/s of fluorescent light). The cells were harvested, and plated onto agar plates of modified F2N agar containing 2.5 µg/mL Zeocin [47].

### Molecular analysis of NsAtWRI1 transformants by genomic PCR, RESDA PCR, and Western blotting

Transfer of the pNsAtWRI1 vector into *N. salina* was confirmed by genomic PCR. The genomic DNA of the cells was extracted using 200 µL of Instagene Matrix (Bio-Rad, USA) at 56 °C for 20 min and at 100 °C for 8 min. The mixture was centrifuged, and the supernatant was used as the template for genomic PCR. Genomic PCR was carried out using the W1 (forward) and W2 (reverse) primers to detect *AtWRI1* and the SR6 (forward) and SR9 (reverse) primers to detect *18S rDNA* (Additional file 1: Table S1) [59] and Ex taq polymerase (TaKaRa, Japan). The PCR amplification was conducted under the following conditions; 95 °C for 5 min, 30 cycles of 95 °C for 1 min, 60 °C for 1 min, 72 °C for 1 min, and then 72 °C for 10 min. The PCR-amplified bands of *AtWRI1* and *18S rDNA* PCR were detected at 1.3 kb and 380 bp, respectively.

RESDA PCR was performed to determine if the transgene had integrated into genomic DNA [60]. RESDA PCR consists of two steps, amplification I (Amp I) and amplification II (Amp II). The Amp I was performed using RESDA fwd 1 primer and three degenerate primers (DegClaI, DegNdeI, DegSspI) (Additional file 1: Table S1) under the following conditions; 96 °C for 5 min, 20 cycles of 95 °C for 1 min, 60 °C for 1 min, 72 °C for 3 min, 10 cycles of 95 °C for 1 min, 40 °C for 1 min, and then 72 °C for 10 min. The Amp II was performed using the RESDA fwd 2 and Q0 primers, and, as a template, 1 µL of the PCR product from the first amplification (Additional file 1: Table S1). The amplification was performed under the following conditions; 96 °C for 5 min, 35 cycles of 95 °C for 1 min, 60 °C for 1 min, 72 °C for 3 min, and then, 72 °C for 10 min. The PCR product from Amp II step was purified and sequenced (Additional file 2: Figure S1).

Western blotting was performed to confirm expression of FLAG-tagged AtWRI1. Protein was extracted from 3 × 10^8^ cells by vigorous mixing with 1.5× modified Laemmli sample buffer [62.5 mM Tris–HCl, pH 7.6, 7% sodium dodecyl sulfate (SDS), 25% glycerol, 5% β-mercaptoethanol, and 0.02% bromophenol blue], followed by incubation at 100 °C for 5 min [47]. The samples were centrifuged, and 10 µL of each supernatant was electrophoresed in SDS-polyacrylamide gels (PAGE) (Any kD Mini-PROTEAN TGX Precast Protein Gels, Bio-Rad, USA), followed by transfer to polyvinylidene difluoride (PVDF) membranes using the Trans-Blot Turbo system (Bio-Rad, USA). The membranes were blocked by incubation with 5% skim milk and 0.1% Tween 20 in phosphate-buffered saline (PBS) and immunoblotted by incubation in a 1:1000 dilution of rabbit anti-FLAG-tag antibody (Cell Signaling Technology, USA) and a 1:5000 dilution of rabbit anti-AtpB antibody (Agrisera, Sweden) for loading control. After washing, the membranes were incubated with a 1:1000 dilution of horseradish peroxidase (HRP)-conjugated anti-rabbit secondary antibody (Cell Signaling Technology, USA). FLAG-tagged AtWRI1 was detected by enhanced chemiluminescence (ECL) reagents and the ChemiDoc system (Bio-Rad).

### Growth analysis of NsAtWRI1 transformants

WT and NsAtWRI1 were cultivated under normal, N limitation, and osmotic stress conditions. Cell growth was analyzed by measuring cell density, specific growth rate, and dry cell weight (DCW). Cell density was estimated by the Cellometer Auto X4 Cell Counter (Nexcelom Bioscience, USA). Specific growth rate was calculated from cell density using the equation:

$${\text{Specific growth rate }} ( {\upmu /{\text{day}}}) = \ln ( {X_{2} /X_{1} } )/(t_{2} - t_{1} ),$$

where *X*_1_ and *X*_2_ are the initial and final cell concentrations and *t*_1_ and *t*_2_ are the first and last days of culture. To determine DCW, cells were filtered with GF/C filter paper (Whatman, USA), washed with deionized water and dried at 105 °C overnight, after which cell weight was measured.

### Total lipid analysis by gravimetric method and HPLC

Folch’s method was used to extract total lipids [61]. Briefly, microalgal cells were harvested, and then lyophilized in a freeze drier (FD5508, IlShin BioiBase, South Korea). A chloroform–methanol mixture (2:1, v/v) was added to the lyophilized biomass and then sonicated for 1 h at room temperature. Deionized water was added to the samples and vortexed vigorously for 5 min. After centrifugation at 3500 rpm for 15 min, the separated lower layer (organic phase) was obtained and filtered using 0.20 μm RC-membrane syringe filters (Sartorius Stedim Biotech, Germany). Total lipid contents were calculated by measuring difference of lipid mass after evaporation of the chloroform. Lipid composition and their contents were determined by High Performance Liquid Chromatography (HPLC) Agilent 1260 and ELSD (Agilent technologies, USA) with a Chromolith^®^Performance-Si ((100 × 4.6 mm I.D.) column (VWR, USA). A gradient system was modified from a previous report [62].

### Fatty acid methyl ester (FAME) analysis

Ten mg of lyophilized biomass was vigorously mixed with a chloroform–methanol mixture (2:1, v/v) for lipid extraction. Then, 0.5 mg of heptadecanoic acid (C17:0) was added as an internal standard. For transesterification, 1 mL of methanol and 300 μL of sulfuric acid were added to the samples, and incubated at 100 °C for 20 min. Each sample was then mixed with 1 mL of deionized water, followed by centrifugation to separate the organic (lower) phase, which was obtained by filtration using 0.20 μm RC-membrane syringe filters (Sartorius Stedim Biotech, Germany). FAMEs were analyzed using a gas chromatograph (GC) (HP 6890, Agilent, USA) equipped with a flame ionized detector (FID) and an HP-INNOWax polyethylene glycol column (HP 19091N-213, Agilent, USA). The oven temperature of the GC was increased from 50 to 250 °C at a rate of 15 °C per min. The FAME contents in these samples were determined by reference to a 37-component mix of FAME standards (F.A.M.E. MIX C8-C24, Supelco, USA).

### Identification of AtWRI1 targets and analysis of RNA expression by qRT-PCR

To select target genes containing AW-boxes [CnTnG(n)_7_CG], where ‘*n*’ represents any nucleotide [28], sequence patterns were predicted with RSAT ‘matrix-scan’ [48]. A total of 7494 sequence patterns that included AW-boxes were detected in the *N. salina* genome. We then obtained 7435 predicted promoter sequences located 500 bp upstream from the start codon of individual genes. Through this process, 475 genes containing AW-boxes in their promoter sequences were identified. These 475 genes could be classified by GO term enrichment test using Blast2GO software with Fisher’s exact test and *P* value < 0.05 (Additional file 6: Figure S3) [63]. The seven genes related to lipid metabolism (*PPDK, LPGAT1, PDH, LPL, DGAT, TAGL,* and *DAGK*) were selected for further experiments. To understand specific function of the seven genes, we repeated enrichment test using Blast2GO software with Fisher’s exact test and *P* value < 0.09 (Table 2; Additional file 7: Figure S4).

The expression levels of these selected target genes were analyzed by qRT-PCR in WT and NsAtWRI1. Cells were incubated for 1 and 8 days under normal, N limitation and osmotic stress conditions. The cells were harvested and RNA was extracted using NucleoZol reagent (Macherey–Nagel, Germany), according to the manufacturer’s protocol. DNA was removed from these RNA samples using DNA-free™ DNase kits (Ambion, USA), and the RNAs were reverse transcribed to cDNA using Superscript™ III Reverse Transcriptase (Invitrogen, USA) and an oligo (dT)_20_ primer (Invitrogen, USA) [47].

qRT-PCR was performed using the CFX96 Real-Time system (Bio-Rad, USA). Used primers for the seven selected target genes and actin gene, used as a loading control for normalization, are summarized in Additional file 1: Table S1. Each 20 μL reaction volume contained 2 μL of cDNA (representing 20 ng of total RNA), 0.5 μL of each forward and reverse primer (at concentrations of 10 μM), 7 μL of distilled water, and 10 μL of Universal SYBR Supermix (Bio-Rad, USA). The qRT-PCR was performed under the following conditions; 95 °C for 2 min; 40 cycles of denaturation at 95 °C for 10 s, annealing at 60 °C for 10 s, and extension at 72 °C for 20 s, followed by denaturation at 95 °C for 10 s and a final melting step at 65–95 °C. Gene expression level was analyzed by the 2^−∆∆Ct^ method, and statistical significance was assessed by Student’s t test [47].

## Additional files

**Additional file 1: Table S1.** Primers used in this study.

**Additional file 2: Figure S1.** RESDA PCR of NsAtWRI1 transformants.

**Additional file 3: Figure S2.** Amino acid sequences of *N. salina* AP2 domain containing proteins.

**Additional file 4: Table S2.** Comparison of NsAP2 transcription factors with AtWRI1.

**Additional file 5: Table S3.** Specific growth rate and biomass yield of WT and NsAtWRI1 transformants.

**Additional file 6: Figure S3.** GO annotation of *N. salina* genes containing AW-boxes in their promoter region in *N. salina*.

**Additional file 7: Figure S4.** Schematic of *N. salina* lipid synthesis-related genes containing AW-boxes in their promoters.

**Additional file 8: Figure S5.** Carbon fixation and lipid synthesis pathway.

**Additional file 9: Figure S6.** Expression profiles of AtWRI1-regulated candidate genes involved in lipid synthesis in NsAtWRI1 2-3 and *N. salina* WT.

## Abbreviations

TF: transcription factor
AtWRI1: *Arabidopsis thaliana* Wrinkled1
qRT-PCR: quantitative real-time polymerase chain reaction
RESDA PCR: restriction enzyme site-directed polymerase chain reaction
DCW: dry cell weight
FAME: fatty acid methyl ester
PPDK: pyruvate phosphate dikinase
LPGAT1: lysophosphatidylglycerol acyltransferase 1
PDH: pyruvate dehydrogenase
LPL: lysophospholipase
DGAT: diacylglycerol acyltransferase family protein
TAGL: triacylglycerol lipase
DAGK: diacylglycerol kinase
GPDH: glycerol-3-phosphate dehydrogenase
GPAT: glycerol-3-phosphate acyltransferase
LPAAT: lysophosphatidic acid acyltransferase
PAP: phosphatidic acid phosphatase
PLA: phospholipase
PLC: phospholipase C
LPL: lysophospholipase
PEPC: phosphoenolpyruvate carboxylase
MDH: malatdehydrogenase
ME: malatenzyme
RubisCO: ribulose-1,5-bisphosphate carboxylase/oxygenase
DHAP: dihydroxyacetone phosphate
G3P: glycerol-3-phosphate
Lyso-PA: lysophosphatidic acid
PA: phosphatidic acid
DAG: diacylglycerol
TAG: triacylglycerol
PC: phosphatidylcholine
Lyso-PC: phosphatidylcholine
G3PC: glycerol-3-phosphocholine
Lyso-PG: lysophophatidylglycerol
PG: phophatidylglycerol
3PGA: 3-phosphoglycerate
PEP: phosphoenolpyruvate
OAA: oxaloacetic acid
MA: malate
MGDG: monogalactosyldiacylglycerol
DGDG: digalactosyldiacylglycerol
PI: phosphatidylinositol

## References

[1] Yen HW, Hu IC, Chen CY, Ho SH, Lee DJ, Chang JS (2013). Microalgae-based biorefinery—from biofuels to natural products. Bioresour Technol.

[2] Scott SA, Davey MP, Dennis JS, Horst I, Howe CJ, Lea-Smith DJ (2010). Biodiesel from algae: challenges and prospects. Curr Opin Biotechnol.

[3] Williams PJlB, Laurens LML (2010). Microalgae as biodiesel & biomass feedstocks: Review & analysis of the biochemistry, energetics & economics. Energy Environ Sci.

[4] Ho SH, Ye X, Hasunuma T, Chang JS, Kondo A (2014). Perspectives on engineering strategies for improving biofuel production from microalgae—a critical review. Biotechnol Adv.

[5] Mata TM, Martins AA, Caetano NS (2010). Microalgae for biodiesel production and other applications: a review. Renew Sust Energ Rev.

[6] Radakovits R, Jinkerson RE, Darzins A, Posewitz MC (2010). Genetic engineering of algae for enhanced biofuel production. Eukaryot Cell.

[7] Niu YF, Wang X, Hu DX, Balamurugan S, Li DW, Yang WD (2016). Molecular characterization of a glycerol-3-phosphate acyltransferase reveals key features essential for triacylglycerol production in *Phaeodactylum tricornutum*. Biotechnol Biofuels.

[8] Yao Y, Lu Y, Peng KT, Huang T, Niu YF, Xie WH (2014). Glycerol and neutral lipid production in the oleaginous marine diatom *Phaeodactylum tricornutum* promoted by overexpression of glycerol-3-phosphate dehydrogenase. Biotechnol Biofuels.

[9] Ma YH, Wang X, Niu YF, Yang ZK, Zhang MH, Wang ZM (2014). Antisense knockdown of pyruvate dehydrogenase kinase promotes the neutral lipid accumulation in the diatom *Phaeodactylum tricornutum*. Microb Cell Fact.

[10] Chen CY, Kao AL, Tsai ZC, Chow TJ, Chang HY, Zhao XQ (2016). Expression of type 2 diacylglycerol acyltransferse gene DGTT1 from *Chlamydomonas reinhardtii* enhances lipid production in *Scenedesmus obliquus*. Biotechnol J.

[11] Blatti JL, Michaud J, Burkart MD (2013). Engineering fatty acid biosynthesis in microalgae for sustainable biodiesel. Curr Opin Chem Biol.

[12] Gimpel JA, Henriquez V, Mayfield SP (2015). In metabolic engineering of eukaryotic microalgae: potential and challenges come with great diversity. Front Microbiol.

[13] Courchesne NM, Parisien A, Wang B, Lan CQ (2009). Enhancement of lipid production using biochemical, genetic and transcription factor engineering approaches. J Biotechnol.

[14] Bajhaiya AK, Ziehe Moreira J, Pittman JK (2017). Transcriptional engineering of microalgae: prospects for high-value chemicals. Trends Biotechnol.

[15] Gargouri M, Park JJ, Holguin FO, Kim MJ, Wang H, Deshpande RR (2015). Identification of regulatory network hubs that control lipid metabolism in *Chlamydomonas reinhardtii*. J Exp Bot.

[16] Boyle NR, Page MD, Liu B, Blaby IK, Casero D, Kropat J (2012). Three acyltransferases and nitrogen-responsive regulator are implicated in nitrogen starvation-induced triacylglycerol accumulation in *Chlamydomonas*. J Biol Chem.

[17] Bajhaiya AK, Dean AP, Zeef LA, Webster RE, Pittman JK (2016). PSR1 Is a global transcriptional regulator of phosphorus deficiency responses and carbon storage metabolism in *Chlamydomonas reinhardtii*. Plant Physiol.

[18] Ngan CY, Wong CH, Choi C, Yoshinaga Y, Louie K, Jia J (2015). Lineage-specific chromatin signatures reveal a regulator of lipid metabolism in microalgae. Nat Plants.

[19] Schulz-Raffelt M, Chochois V, Auroy P, Cuine S, Billon E, Dauvillee D (2016). Hyper-accumulation of starch and oil in a *Chlamydomonas* mutant affected in a plant-specific DYRK kinase. Biotechnol Biofuels.

[20] Rubio V, Linhares F, Solano R, Martin AC, Iglesias J, Leyva A (2001). A conserved MYB transcription factor involved in phosphate starvation signaling both in vascular plants and in unicellular algae. Genes Dev.

[21] Shaw LM, McIntyre CL, Gresshoff PM, Xue GP (2009). Members of the Dof transcription factor family in *Triticum aestivum* are associated with light-mediated gene regulation. Funct Integr Genom.

[22] Papi M, Sabatini S, Altamura MM, Hennig L, Schafer E, Costantino P (2002). Inactivation of the phloem-specific Dof zinc finger gene DAG1 affects response to light and integrity of the testa of Arabidopsis seeds. Plant Physiol.

[23] Yanagisawa S (2000). Dof1 and Dof2 transcription factors are associated with expression of multiple genes involved in carbon metabolism in maize. Plant J.

[24] Wang HW, Zhang B, Hao YJ, Huang J, Tian AG, Liao Y (2007). The soybean Dof-type transcription factor genes, GmDof4 and GmDof11, enhance lipid content in the seeds of transgenic *Arabidopsis* plants. Plant J..

[25] Zhang J, Hao Q, Bai L, Xu J, Yin W, Song L (2014). Overexpression of the soybean transcription factor GmDof4 significantly enhances the lipid content of *Chlorella ellipsoidea*. Biotechnol Biofuels.

[26] Ibanez-Salazar A, Rosales-Mendoza S, Rocha-Uribe A, Ramirez-Alonso JI, Lara-Hernandez I, Hernandez-Torres A (2014). Over-expression of Dof-type transcription factor increases lipid production in *Chlamydomonas reinhardtii*. J Biotechnol.

[27] Baud S, Mendoza MS, To A, Harscoet E, Lepiniec L, Dubreucq B (2007). WRINKLED1 specifies the regulatory action of LEAFY COTYLEDON2 towards fatty acid metabolism during seed maturation in *Arabidopsis*. Plant J..

[28] Maeo K, Tokuda T, Ayame A, Mitsui N, Kawai T, Tsukagoshi H (2009). An AP2-type transcription factor, WRINKLED1, of *Arabidopsis thaliana* binds to the AW-box sequence conserved among proximal upstream regions of genes involved in fatty acid synthesis. Plant J..

[29] Shen B, Allen WB, Zheng P, Li C, Glassman K, Ranch J (2010). Expression of ZmLEC1 and ZmWRI1 increases seed oil production in maize. Plant Physiol.

[30] Focks N, Benning C (1998). wrinkled1: a novel, low-seed-oil mutant of *Arabidopsis* with a deficiency in the seed-specific regulation of carbohydrate metabolism. Plant Physiol.

[31] Ma XN, Chen TP, Yang B, Liu J, Chen F (2016). Lipid Production from *Nannochloropsis*. Marine drugs.

[32] Shih CH, Chen HY, Lee HC, Tsai HJ (2015). Purple chromoprotein gene serves as a new selection marker for transgenesis of the microalga *Nannochloropsis oculata*. PLoS ONE.

[33] Kang NK, Lee B, Shin SE, Jeon S, Park MS, Yang JW (2015). Use of conditioned medium for efficient transformation and cost-effective cultivation of *Nannochloropsis salina*. Bioresour Technol.

[34] Li F, Gao D, Hu H (2014). High-efficiency nuclear transformation of the oleaginous marine *Nannochloropsis* species using PCR product. Biosci Biotechnol Biochem.

[35] Kilian O, Benemann CS, Niyogi KK, Vick B (2011). High-efficiency homologous recombination in the oil-producing alga *Nannochloropsis* sp. Proc Natl Acad Sci USA.

[36] Kang NK, Choi GG, Kim EK, Shin SE, Jeon S, Park MS (2015). Heterologous overexpression of sfCherry fluorescent protein in *Nannochloropsis salina*. Biotechnol Rep.

[37] Wang D, Ning K, Li J, Hu J, Han D, Wang H (2014). *Nannochloropsis* genomes reveal evolution of microalgal oleaginous traits. PLoS Genet.

[38] Zheng M, Tian J, Yang G, Zheng L, Chen G, Chen J (2013). Transcriptome sequencing, annotation and expression analysis of *Nannochloropsis* sp. at different growth phases. Gene.

[39] Radakovits R, Jinkerson RE, Fuerstenberg SI, Tae H, Settlage RE, Boore JL (2012). Draft genome sequence and genetic transformation of the oleaginous alga *Nannochloropis gaditana*. Nat Commun.

[40] Vieler A, Wu G, Tsai CH, Bullard B, Cornish AJ, Harvey C (2012). Genome, functional gene annotation, and nuclear transformation of the heterokont oleaginous alga *Nannochloropsis oceanica* CCMP1779. PLoS Genet.

[41] Corteggiani Carpinelli E, Telatin A, Vitulo N, Forcato C, D’Angelo M, Schiavon R (2014). Chromosome scale genome assembly and transcriptome profiling of *Nannochloropsis gaditana* in nitrogen depletion. Mol plant.

[42] Starkenburg SR, Kwon KJ, Jha RK, McKay C, Jacobs M, Chertkov O (2014). A pangenomic analysis of the *Nannochloropsis* organellar genomes reveals novel genetic variations in key metabolic genes. BMC Genom.

[43] Beacham TA, Ali ST (2016). Growth dependent silencing and resetting of DGA1 transgene in *Nannochloropsis salina*. Algal Res.

[44] Kaye Y, Grundman O, Leu S, Zarka A, Zorin B, Didi-Cohen S (2015). Metabolic engineering toward enhanced LC-PUFA biosynthesis in *Nannochloropsis oceanica*: overexpression of endogenous Delta 12 desaturase driven by stress-inducible promoter leads to enhanced deposition of polyunsaturated fatty acids in TAG. Algal Res.

[45] Iwai M, Hori K, Sasaki-Sekimoto Y, Shimojima M, Ohta H (2015). Manipulation of oil synthesis in *Nannochloropsis* strain NIES-2145 with a phosphorus starvation-inducible promoter from *Chlamydomonas reinhardtii*. Front Microbiol.

[46] Hu J, Wang D, Li J, Jing G, Ning K, Xu J (2014). Genome-wide identification of transcription factors and transcription-factor binding sites in oleaginous microalgae *Nannochloropsis*. Sci Rep.

[47] Kang NK, Jeon S, Kwon S, Koh HG, Shin SE, Lee B (2015). Effects of overexpression of a bHLH transcription factor on biomass and lipid production in *Nannochloropsis salina*. Biotechnol Biofuels.

[48] Turatsinze JV, Thomas-Chollier M, Defrance M, van Helden J (2008). Using RSAT to scan genome sequences for transcription factor binding sites and cis-regulatory modules. Nat Protoc.

[49] Baud S, Wuilleme S, To A, Rochat C, Lepiniec L (2009). Role of WRINKLED1 in the transcriptional regulation of glycolytic and fatty acid biosynthetic genes in *Arabidopsis*. Plant J.

[50] Barka F, Angstenberger M, Ahrendt T, Lorenzen W, Bode HB, Buchel C (2016). Identification of a triacylglycerol lipase in the diatom *Phaeodactylum tricornutum*. Biochim Biophys Acta.

[51] Guarnieri MT, Nag A, Smolinski SL, Darzins A, Seibert M, Pienkos PT (2011). Examination of triacylglycerol biosynthetic pathways via de novo transcriptomic and proteomic analyses in an unsequenced microalga. PLoS ONE.

[52] Merida I, Avila-Flores A, Merino E (2008). Diacylglycerol kinases: at the hub of cell signalling. Biochem J.

[53] Terashima M, Specht M, Hippler M (2011). The chloroplast proteome: a survey from the *Chlamydomonas reinhardtii* perspective with a focus on distinctive features. Curr Genet.

[54] Chastain CJ, Failing CJ, Manandhar L, Zimmerman MA, Lakner MM, Nguyen TH (2011). Functional evolution of C(4) pyruvate, orthophosphate dikinase. J Exp Bot.

[55] Shindou H, Hishikawa D, Harayama T, Eto M, Shimizu T (2013). Generation of membrane diversity by lysophospholipid acyltransferases. J Biochem.

[56] Li J, Han D, Wang D, Ning K, Jia J, Wei L (2014). Choreography of transcriptomes and lipidomes of *Nannochloropsis* reveals the mechanisms of oil synthesis in microalgae. Plant Cell.

[57] Jinkerson RE, Radakovits R, Posewitz MC (2013). Genomic insights from the oleaginous model alga *Nannochloropsis gaditana*. Bioengineered.

[58] Gibson DG, Young L, Chuang RY, Venter JC, Hutchison CA, Smith HO (2009). Enzymatic assembly of DNA molecules up to several hundred kilobases. Nat Methods.

[59] Nakayama T, Watanabe S, Mitsui K, Uchida H, Inouye I (1996). The phylogenetic relationship between the *Chlamydomonadales* and *Chlorococcales* inferred from 18SrDNA sequence data. Phycol Res.

[60] Gonzalez-Ballester D, de Montaigu A, Galvan A, Fernandez E (2005). Restriction enzyme site-directed amplification PCR: a tool to identify regions flanking a marker DNA. Anal Biochem.

[61] Folch J, Lees M, Sloane Stanley GH (1957). A simple method for the isolation and purification of total lipides from animal tissues. J Biol Chem.

[62] Graeve M, Janssen D (2009). Improved separation and quantification of neutral and polar lipid classes by HPLC-ELSD using a monolithic silica phase: application to exceptional marine lipids. J Chromatogr B Analyt Technol Biomed Life Sci.

[63] Conesa A, Gotz S (2008). Blast2GO: a comprehensive suite for functional analysis in plant genomics. Int J Plant Genom.

