# Supplementary material for: Increased lipid production by heterologous expression of AtWRI1 transcription factor in Nannochloropsis salina
Source: Biotechnol Biofuels. 2017 Oct 10;10:231. doi: 10.1186/s13068-017-0919-5 (PMC5635583; doi:10.1186/s13068-017-0919-5)
Supplement: Supplementary file 1 — Additional file 1: Table S1. Primers used in this study. [file 13068_2017_919_MOESM1_ESM.docx]

**Table S1 Primers used in this study**

| Primer | Sequence 5'-3' | Purpose |
| --- | --- | --- |
| W1 | CAAGAAGCTGTCTTTTTGTGAAGCATGAAGAAACGGCTGACGACCTC | Amplifying AtWRI1 cDNA for Gibson assembly |
| W2 | AAAAGTGGTCGGACAAAAGGAGTTTCACTTGTCGTCGTCGTCCTTGTAGTCCACCAAATAATTACAGCTGACGGACGT |  |
| W3 | GACTACAAGGACGACGACGACAAGTGAAACTCCTTTTGTCCGACCACTTTTACAATC | Amplifying pNsAtWRI1 backbone for Gibson assembly |
| W4 | GGAGGTCGTCAGCCGTTTCTTCATGCTTCACAAAAAGACAGCTTCTTGATATTGAC |  |
| SR6 | GTCAGAGGTGAAATTCTTGG | 18s rDNA |
| SR9 | AACTAAGAACGGCATGCAC |  |
| qTAGL fwd | CGAAGAGAGCAACGGCGATA | qRT-PCR for TAGL mRNA |
| qTAGL rev | CCGCGAAAGTTGCCTGATTC |  |
| qDAGK fwd | TCTTGGTCACGATTGGTCGG | qRT-PCR for DAGK mRNA |
| qDAGK rev | GCCTCTGTCATGCGCAATTC |  |
| qLPL fwd | TGTCAGGGAATGGCTGGATG | qRT-PCR for LPL mRNA |
| qLPL rev | CACTCACACCGACTCTCGTC |  |
| qLPGAT1 fwd | TGTACGACGCTCCCTTCAAC | qRT-PCR for LPGAT1 mRNA |
| qLPGAT1 rev | GTTGGTTCAGCCAATCCTGC |  |
| qDGAT fwd | CCGCACGGTCTCTTCACATA | qRT-PCR for DGAT mRNA |
| qDGAT rev | CACCGCGTTTGGTTTCATGT |  |
| qPDH fwd | GAGCCATGACCCACCACATT | qRT-PCR for PDH mRNA |
| qPDH rev | ACCACGTCCCCGTTTTTGAT |  |
| qPPDK fwd | GCGGGAGATTTTCTCCGTCA | qRT-PCR for PPDK mRNA |
| qPPDK rev | TTCCAGCGCCTTGATCTTGT |  |
| qACTIN fwd | GTGTTTCCCTCCATCGTG | qRT-PCR for Actin mRNA |
| qACTIN rev | CCAGTTCGTCACAATACCG |  |
| RESDA fwd1 | AAGAGACAGGATGAGGATCG | RESDA Amp I |
| DegClaI | CCAGTGAGCAGAGTGACG IIIIINNS ATCGAT W |  |
| DegNdeI | CCAGTGAGCAGAGTGACG IIIIINNS CATATG W |  |
| DegSspI | CCAGTGAGCAGAGTGACG IIIIINNS AATATT W |  |
| RESDA fwd2 | TGTCATCCCACCTTGCTCCT | RESDA Amp II |
| Q0 | CCAGTGAGCAGAGTGACG |  |
