# Supplementary material for: Increased lipid production by heterologous expression of AtWRI1 transcription factor in Nannochloropsis salina
Source: Biotechnol Biofuels. 2017 Oct 10;10:231. doi: 10.1186/s13068-017-0919-5 (PMC5635583; doi:10.1186/s13068-017-0919-5)
Supplement: Supplementary file 2 — Additional file 2: Figure S1. RESDA PCR of NsAtWRI1 transformants. [file 13068_2017_919_MOESM2_ESM.docx]

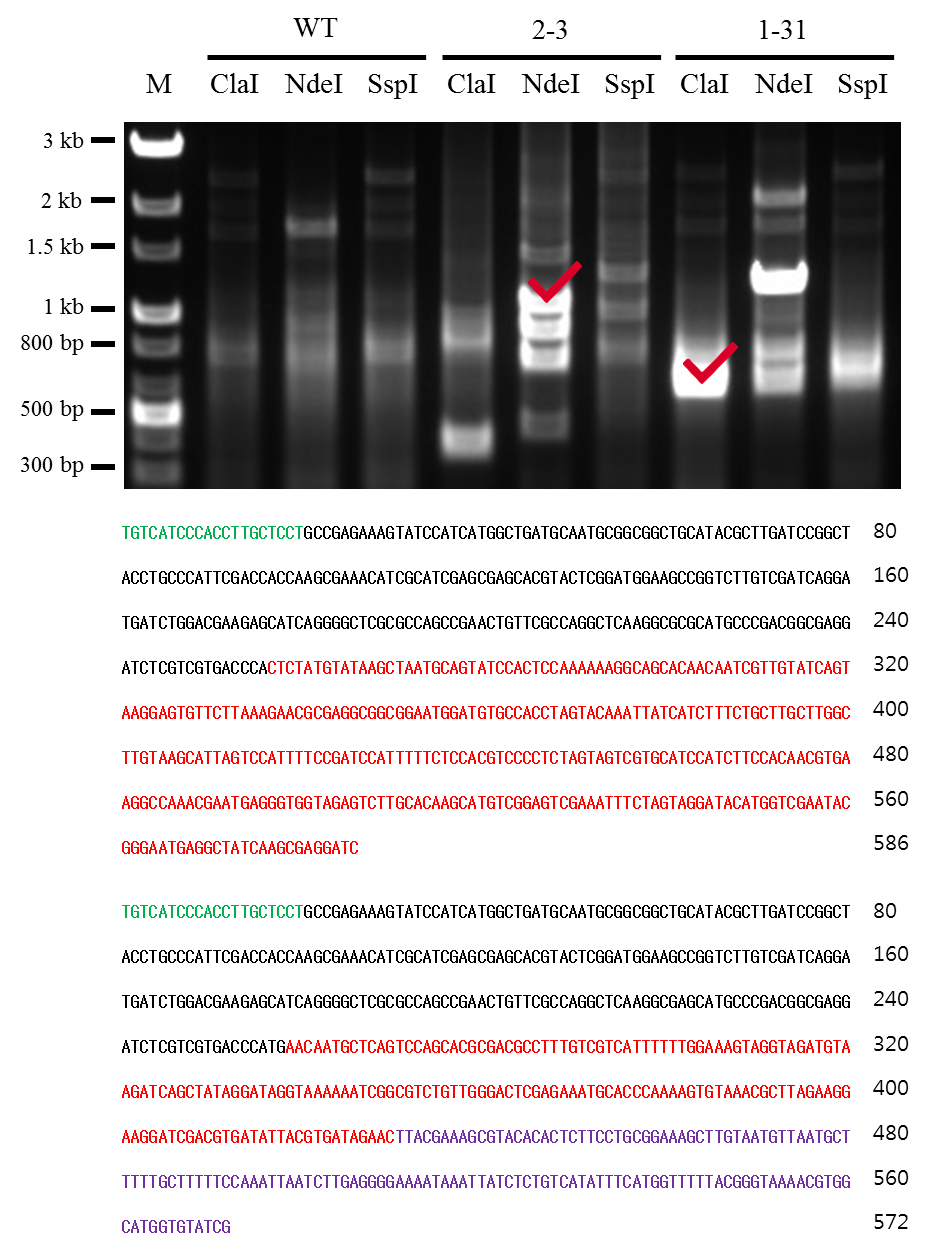


**a**

**b**

**c**

**Figure S1.** RESDA PCR of NsAtWRI1 transformants. **a** Results of the Amp II. The red marked band in the gel was recovered and sequenced. **b** Determined sequence of the integration site in NsAtWRI1 2-3. The integration site was near beta-tubulin (homologous to Naga_10009g86 in *N. gaditana* B-31). **c** Integration sequence of NsAtWRI1 1-31. The integration site was near hypothetical protein (homologous to Naga_100450g4 in *N. gaditana* B-31). Integration sites were found in *N. gaditana* B-31 by homology search with *N. salina* using *Naanochloprsis* genome portal (<http://www.Nannochloropsis.org/>). The purple letters represent CDS of hypothetical protein. The green, black, and red letters represent sequences of the RESDA fwd2 primer, the vector, and genomic DNA, respectively. *M*, marker; *WT* wild type.
