# Supplementary material for: Increased lipid production by heterologous expression of AtWRI1 transcription factor in Nannochloropsis salina
Source: Biotechnol Biofuels. 2017 Oct 10;10:231. doi: 10.1186/s13068-017-0919-5 (PMC5635583; doi:10.1186/s13068-017-0919-5)
Supplement: Supplementary file 3 — Additional file 3: Figure S2. Amino acid sequences of N. salina AP2 domain containing proteins. [file 13068_2017_919_MOESM3_ESM.docx]

**a**

**b**

**c**


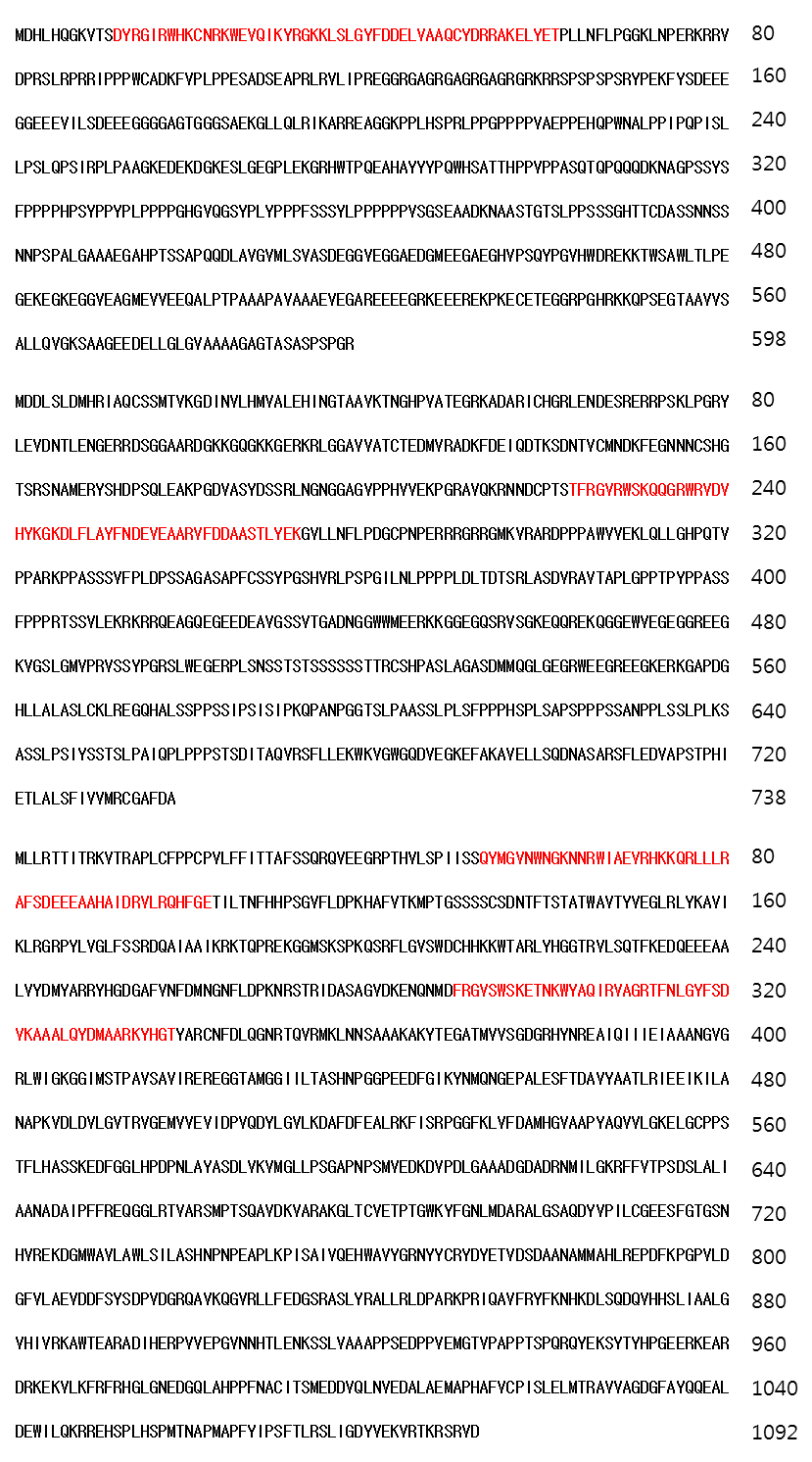


**Figure S2.** Amino acid sequences of *N. salina* AP2 domain containing proteins. **a** NsAP2-1, **b** NsAP2-2, and **c** NsAP2-3. Red letters represent the AP2 domain. AP2 domains were determined by using Pfam database.
