# Supplementary material for: Increased lipid production by heterologous expression of AtWRI1 transcription factor in Nannochloropsis salina
Source: Biotechnol Biofuels. 2017 Oct 10;10:231. doi: 10.1186/s13068-017-0919-5 (PMC5635583; doi:10.1186/s13068-017-0919-5)
Supplement: Supplementary file 4 — Additional file 4: Table S2. Comparison of NsAP2 transcription factors with AtWRI1. [file 13068_2017_919_MOESM4_ESM.docx]

**Table S2 Comparison of NsAP2 transcription factors with AtWRI1.**

| Protein name | Blast score (bits)^a^ | Blast E-value^a^ | Identity (%)^b^ |
| --- | --- | --- | --- |
| AtWRI1 | 633 | 0.0 |  |
| NsAP2-1 | 45 | 5E-08 | 8.3 |
| NsAP2-2 | 32 | 3E-04 | 7.5 |
| NsAP2-3 | 31 | 9E-04 | 6.95 |

^a^Blast scores and E-value were determined by BLASTP algorithm in NCBI website (https://blast.ncbi.nlm.nih.gov/Blast.cgi).

^b^Percentages of amino acid sequence were compared between NsAP2 TFs and AtWRI1 by pairwise alignment using CLC Main Workbench (CLC bio, Aarhus, Denmark).
