# Supplementary material for: Increased lipid production by heterologous expression of AtWRI1 transcription factor in Nannochloropsis salina
Source: Biotechnol Biofuels. 2017 Oct 10;10:231. doi: 10.1186/s13068-017-0919-5 (PMC5635583; doi:10.1186/s13068-017-0919-5)
Supplement: Supplementary file 5 — Additional file 5: Table S3. Specific growth rate and biomass yield of WT and NsAtWRI1 transformants. [file 13068_2017_919_MOESM5_ESM.docx]

**Table S3 Specific growth rate and biomass yield of WT and NsAtWRI1 transformants**

| Culture condition | Strain | Maximum specific | Biomass yield (g/L) | |
| --- | --- | --- | --- | --- |
|  |  | growth rate (day^-1^) | 8 day | 12 day |
| Normal | WT | 0.45 ± 0.10 | 1.22 ± 0.40 | 2.05 ± 0.21 |
|  | 2-3 | 0.57 ± 0.09 | 1.50 ± 0.45 | 2.53 ± 0.28* |
|  | 1-31 | 0.55 ± 0.10 | 1.36 ± 0.38 | 2.28 ± 0.28 |
| N limitation | WT | 0.49 ± 0.10 | 0.75 ± 0.15 | 1.15 ± 0.28 |
|  | 2-3 | 0.66 ± 0.07* | 0.84 ± 0.13 | 1.15 ± 0.12 |
|  | 1-31 | 0.59 ± 0.08 | 0.81 ± 0.04 | 1.19 ± 0.17 |
| Osmotic stress | WT | 0.54 ± 0.03 | 1.24 ± 0.52 | 2.03 ± 0.31 |
|  | 2-3 | 0.56 ± 0.06 | 1.48 ± 0.39 | 2.23 ± 0.50 |
|  | 1-31 | 0.57 ± 0.06 | 1.35 ± 0.45 | 2.05 ± 0.38 |

The data points represent the average of samples and error bars indicate standard deviation (*n* = 4). Significant differences against WT for the same condition, as determined by Student’s *t* test, are indicated by asterisks (* *P* < 0.05, ** *P* < 0.01, *** *P* < 0.001).
