# Supplementary material for: Increased lipid production by heterologous expression of AtWRI1 transcription factor in Nannochloropsis salina
Source: Biotechnol Biofuels. 2017 Oct 10;10:231. doi: 10.1186/s13068-017-0919-5 (PMC5635583; doi:10.1186/s13068-017-0919-5)
Supplement: Supplementary file 6 — Additional file 6: Figure S3. GO annotation of N. salina genes containing AW-boxes in their promoter region in N. salina. [file 13068_2017_919_MOESM6_ESM.docx]

**Biological process**

**Cellular component**

**Molecular function**

**Figure S3.** GO annotation of genes containing AW-boxes in their promoter region in *N. salina*. The 69 GO-terms were classified into three GO domains (biological processes, cellular components, and molecular function). GO classification was performed using the Blast2GO tool with Fisher’s exact text and P-value < 0.05 (<https://www/blast2go.com/>). Reference set and test set represent *N. salina* whole genes and the genes including AW-box in their promoters, respectively.
