# Supplementary material for: Increased lipid production by heterologous expression of AtWRI1 transcription factor in Nannochloropsis salina
Source: Biotechnol Biofuels. 2017 Oct 10;10:231. doi: 10.1186/s13068-017-0919-5 (PMC5635583; doi:10.1186/s13068-017-0919-5)
Supplement: Supplementary file 7 — Additional file 7: Figure S4. Schematic of N. salina lipid synthesis-related genes containing AW-boxes in their promoters. [file 13068_2017_919_MOESM7_ESM.docx]

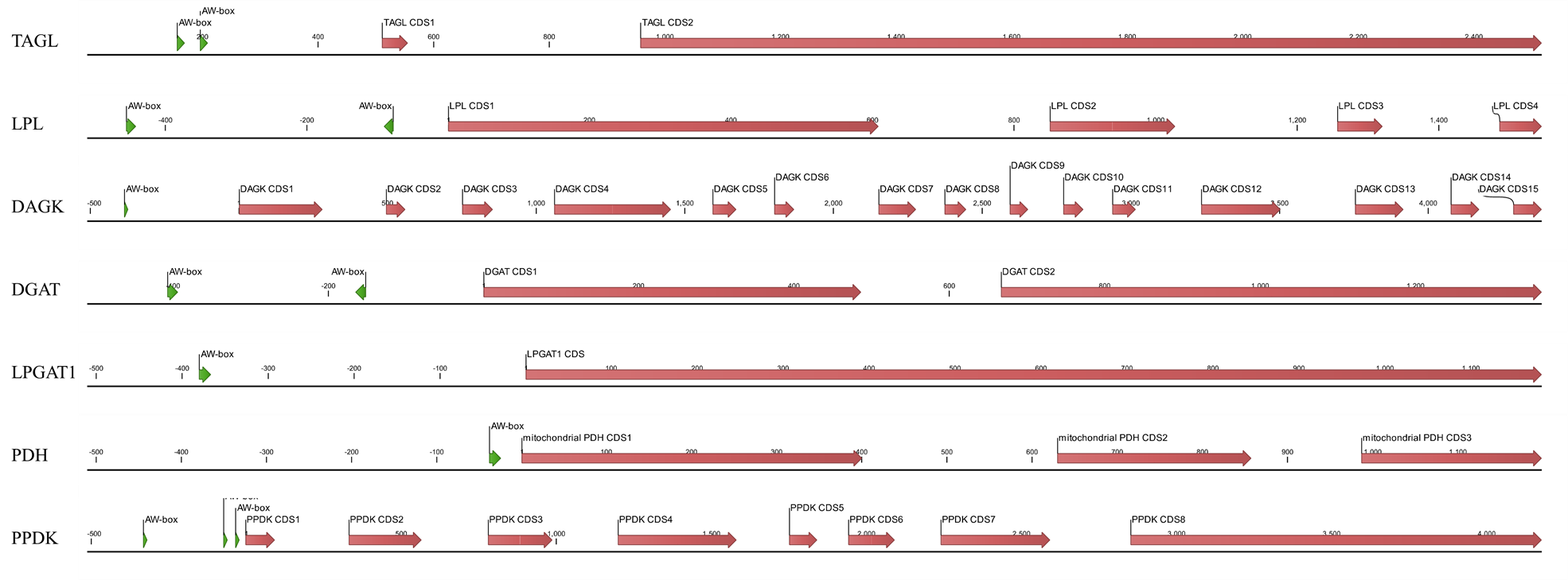


**Figure S4.** Schematic of *N. salina* lipid synthesis-related genes containing AW-boxes in their promoters*.* The green and red arrows represent the AW-box and the CDS, respectively, of each gene.
