# Supplementary material for: Increased lipid production by heterologous expression of AtWRI1 transcription factor in Nannochloropsis salina
Source: Biotechnol Biofuels. 2017 Oct 10;10:231. doi: 10.1186/s13068-017-0919-5 (PMC5635583; doi:10.1186/s13068-017-0919-5)
Supplement: Supplementary file 8 — Additional file 8: Figure S5. Carbon fixation and lipid synthesis pathway. [file 13068_2017_919_MOESM8_ESM.docx]

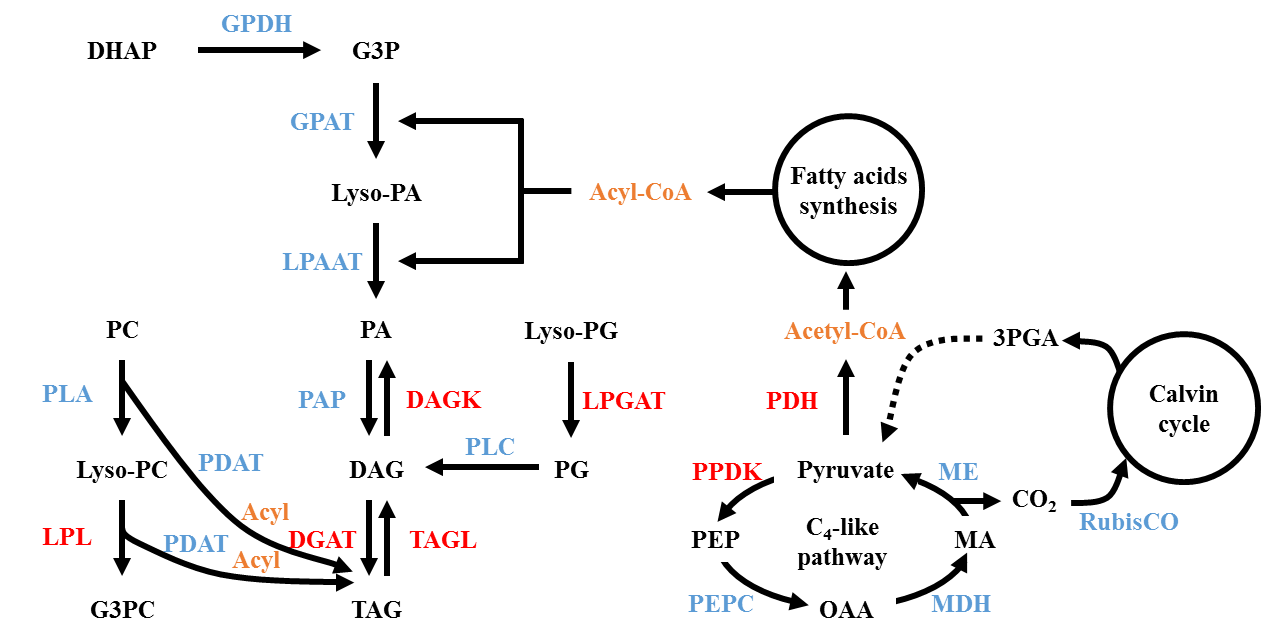


**Figure S5.** Carbon fixation and lipid synthesis pathway. Black, blue, and red letters indicated metabolites, enzymes, and possible AtWRI1-regualted enzymes, respectively. A dotted arrow represents simplified multi-steps in a pathway. Abbreviations: *DHAP* dihydroxyacetone phosphate, *G3P* glycerol-3-phosphate, *Lyso-PA* lysophosphatidic acid, *PA* phosphatidic acid, *DAG* diacylglycerol, *TAG* triacylglycerol, *PC* phosphatidylcholine, *Lyso-PC* phosphatidylcholine, *G3PC* glycerol-3-phosphocholine, *Lyso-PG* lysophophatidylglycerol, *PG* phophatidylglycerol, *3PGA* 3-phosphoglycerate, *PEP* phosphoenolpyruvate, *OAA* oxaloacetic acid, *MA* malate, *GPDH* glycerol-3-phosphate dehydrogenase, *GPAT* glycerol-3-phosphate acyltransferase, *LPAAT* lysophosphatidic acid acyltransferase, *PAP* phosphatidic acid phosphatase, *DGAT* diacylglycerol acyltransferase, *TAGL* triacylglycerol lipase, *DAGK* diacylglycerol kinase, *LPGAT* lysophosphatidylglycerol acyltransferase, *PLA* phospholipase, *PLC* phospholipase C, *LPL* lysophospholipase, *PDH* pyruvate dehydrogenase, *PPDK* pyruvate phosphate dikinase, *PEPC* phosphoenolpyruvate carboxylase, *MDH* malatdehydrogenase, *ME* malatenzyme, *RubisCO* ribulose-1,5-bisphosphate carboxylase/oxygenase.
