# Supplementary material for: Increased lipid production by heterologous expression of AtWRI1 transcription factor in Nannochloropsis salina
Source: Biotechnol Biofuels. 2017 Oct 10;10:231. doi: 10.1186/s13068-017-0919-5 (PMC5635583; doi:10.1186/s13068-017-0919-5)
Supplement: Supplementary file 9 — Additional file 9: Figure S6. Expression profiles of AtWRI1-regulated candidate genes involved in lipid synthesis in NsAtWRI1 2-3 and N. salina WT. [file 13068_2017_919_MOESM9_ESM.docx]

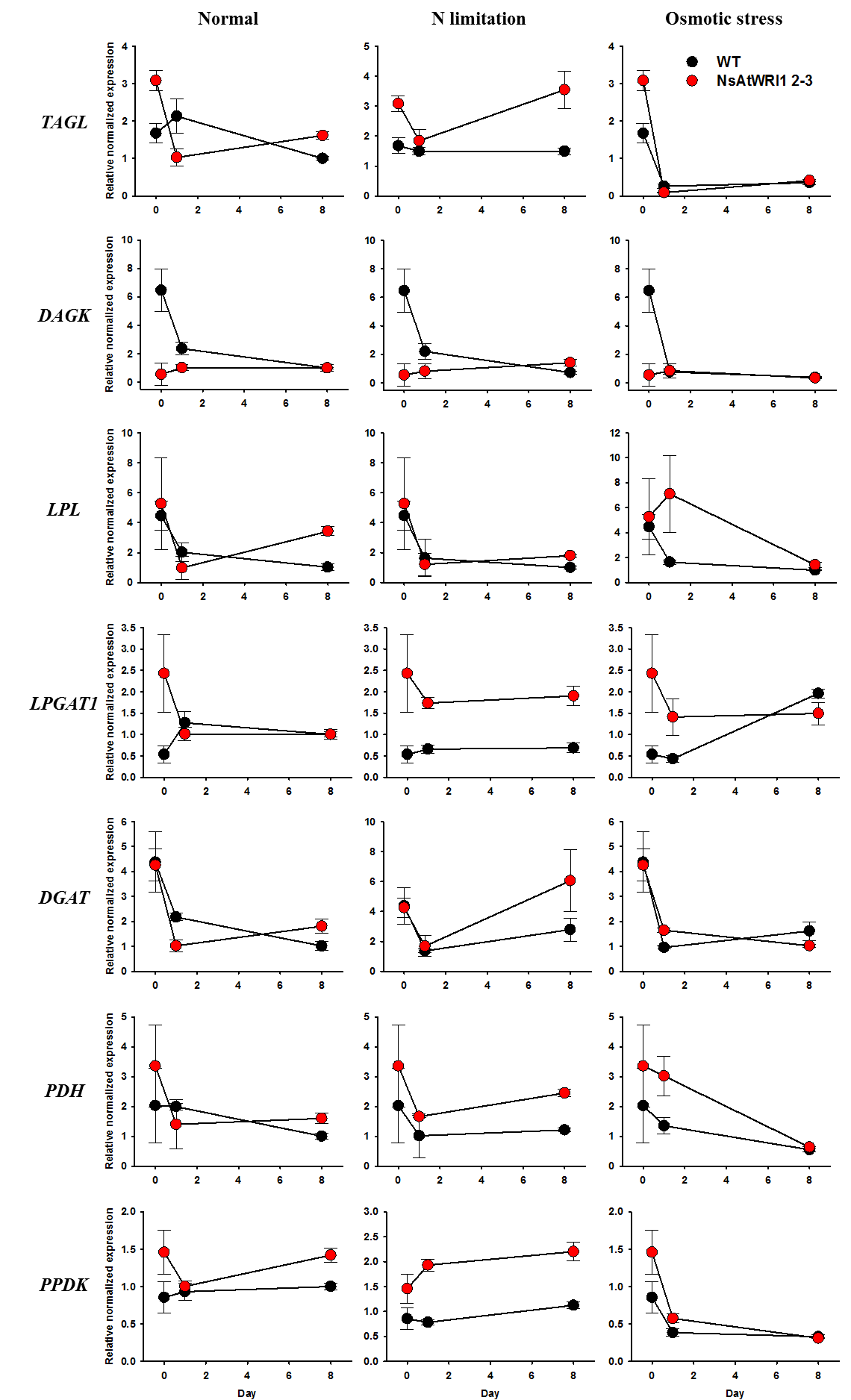


**Figure S6.** Expression profiles of AtWRI1-regulated candidate genes involved in lipid synthesis in NsAtWRI1 2-3 and *N. salina* WT. The expression levels of these genes were determined by qRT-PCR, normalized by that of *actin*. The data points represent the average of samples and error bars indicate standard deviation (n=3). Abbreviations: *TAGL* triacylglycerol lipase, *DAGK* diacylglycerol kinase, *LPL* lysophospholipase, *LPGAT1* lysophosphatidylglycerol acyltransferase 1, *DGAT* diacylglycerol acyltransferase family protein, *PPDK* pyruvate phosphate dikinase, *PDH* dihydrolipoyllysine-residue acetyltransferase component of pyruvate dehydrogenase mitochondrial-like.
